# Analysis of the relationship between coexpression domains and chromatin 3D organization

**DOI:** 10.1371/journal.pcbi.1005708

**Published:** 2017-09-13

**Authors:** María E. Soler-Oliva, José A. Guerrero-Martínez, Valentina Bachetti, José C. Reyes

**Affiliations:** Centro Andaluz de Biología Molecular y Medicina Regenerativa-CABIMER, Consejo Superior de Investigaciones Científicas-Universidad de Sevilla-Universidad Pablo de Olavide (CSIC-USE-UPO), Sevilla, Spain; University of Southern California, UNITED STATES

## Abstract

Gene order is not random in eukaryotic chromosomes, and co-regulated genes tend to be clustered. The mechanisms that determine co-regulation of large regions of the genome and its connection with chromatin three-dimensional (3D) organization are still unclear however. Here we have adapted a recently described method for identifying chromatin topologically associating domains (TADs) to identify coexpression domains (which we term “CODs”). Using human normal breast and breast cancer RNA-seq data, we have identified approximately 500 CODs. CODs in the normal and breast cancer genomes share similar characteristics but differ in their gene composition. COD genes have a greater tendency to be coexpressed with genes that reside in other CODs than with non-COD genes. Such inter-COD coexpression is maintained over large chromosomal distances in the normal genome but is partially lost in the cancer genome. Analyzing the relationship between CODs and chromatin 3D organization using Hi-C contact data, we find that CODs do not correspond to TADs. In fact, intra-TAD gene coexpression is the same as random for most chromosomes. However, the contact profile is similar between gene pairs that reside either in the same COD or in coexpressed CODs. These data indicate that co-regulated genes in the genome present similar patterns of contacts irrespective of the frequency of physical chromatin contacts between them.

## Author summary

Prokaryotic operons normally comprise functionally related genes whose expression is coordinated. Even though operons do not exist in most eukaryotes, results from the last fifteen years indicate that gene order is nonetheless not random in eukaryotes, and that coexpressed genes tend to be grouped in the genome. We identify here about 500 coexpression domain (CODs) in normal breast tissue. Interestingly, we find that genes within CODs often are coexpressed with other genes that reside in other CODs placed very far away in the same chromosome, which is indicative of long-range inter-COD co-regulation. Furthermore, we find that coexpressed genes within CODs or within co-regulated CODs display similar three-dimensional chromatin contacts, suggesting a spatial coordination of CODs.

## Introduction

Genome-wide expression studies have shown that gene order is not random in eukaryotes, and that genes with similar expression patterns are often linked (reviewed in [1, 2]). For instance, early work in yeast showed that neighboring pairs of genes (adjacent and nonadjacent-but-nearby) have correlated expression that is independent of their orientation [3]. Furthermore, Cho et al. showed that 25% of genes with cell cycle–regulated expression patterns were linked to genes induced in the same cell cycle phase [4]. Since then, clusters of genes with similar expression patterns and related functions have been identified in several model organisms. For example, muscle expressed genes have been shown to cluster in *Caenorhabditis elegans* [5] and in humans [6]. Clustering of other tissue-specific genes has been reported in Drosophila [7], mouse [8, 9], and human [10, 11]. Lee and Sonnhameer showed that genes involved in the same KEGG pathway tend to be clustered in several eukaryotic genomes [12]. Kosak and Groudine defined tandem gene arrays as contiguous stretches of genes differentially expressed in the same way during cellular development or differentiation [13]. Tandem gene arrays, mostly constituted by two or three genes co-regulated during hematopoiesis or myogenesis have been described [14, 15]. Other works have suggested the existence of clusters of coexpressed genes with little or absence of co-functionality [16, 17]. Finally, clusters of housekeeping genes [18] or of highly expressed genes [19] have been also reported.

The mechanisms responsible for coexpression of gene clusters are unknown. Coexpression of gene pairs can be explained by bidirectional promoters or by defects in transcription termination. In fact, polycistronic transcripts have been detected in some eukaryotes. For example, *C*. *elegans* contains around 1000 operons that are 2–8 genes long [20]. However, polycistronic transcripts are not common in mammals [21]. It has been proposed that long-range cis regulatory elements, such as enhancers and local control regions, may be responsible to a certain extent for coexpression of gene clusters [2, 22]. So far, this has only been demonstrated for some well-known examples, such as the globin locus or the *Hox* genes. The existence of nuclear and chromatin physical three-dimensional (3D) domains [23, 24] appears an obvious possible mechanism to explain coexpression clusters. Microscopic techniques have demonstrated that chromosomes occupy specific regions within the nucleus, with larger chromosomes positioned closer to the nuclear envelope, and smaller chromosomes generally located in the center of the nucleus [25–27]. In addition, chromosome organization has been demonstrated to be nonrandom as a function of cellular differentiation [28]. At the gene level, several reports have demonstrated that inactive genes are often located close to the nuclear lamina, and that gene activation triggers the movement of loci to the center of the nucleus [29–31]. Furthermore, some microscopy data indicate the existence of transcription factories, in which distant genes appear closely linked [32, 33]. Chromosome conformation capture (3C) and 3C-based technologies (4C, 5C, and Hi-C) have revealed the existence of a vast number of chromatin interactions along the genome (recently reviewed in [23, 34]). Most of the chromatin contacts occur within proximal regions; however, intra-chromosomal long-range contacts and, to a lesser extent, inter-chromosomal contacts also occur [35–37]. Contacts between promoter and transcription termination regions, and between promoters and distant regulatory regions, have been well characterized and occur at the kilobase (kb) to megabase (Mb) scale. However, the functional implications of long-distance interactions are less well understood and have been associated with transcription factories [38, 39]. The existence of regions of high-density of internal contacts surrounded by regions of relatively low density of contacts has suggested that the genome is organized into modular domains, called topologically associating domains (TADs) or contact domains [40–42]. TADs usually show specific patterns of histone marks and, in some cases, a coordinated regulation of their genes [15, 41, 43]. However, the relationship at the genomic level between coexpression gene clusters and TADs has not been studied.

Here we have computationally identified and characterized human coexpression domains (CODs) of normal breast and cancer breast samples. Interestingly, we find that COD genes tend also to be coexpressed with other genes localized in different CODs, indicating an inter-COD co-regulation. As compared to the genome of normal breast tissue, the breast cancer genome had a similar number of CODs but a different COD gene composition, with less co-regulation between CODs, suggesting less structured expression patterns. CODs are not coincident with TADs or contacts domains. However, we observed a similar profile of long-range chromatin contacts between co-regulated CODs, indicating that co-regulated CODs interact with similar regions of the genome.

## Results

### Identification of coexpression domains (CODs)

**Fig 1:**
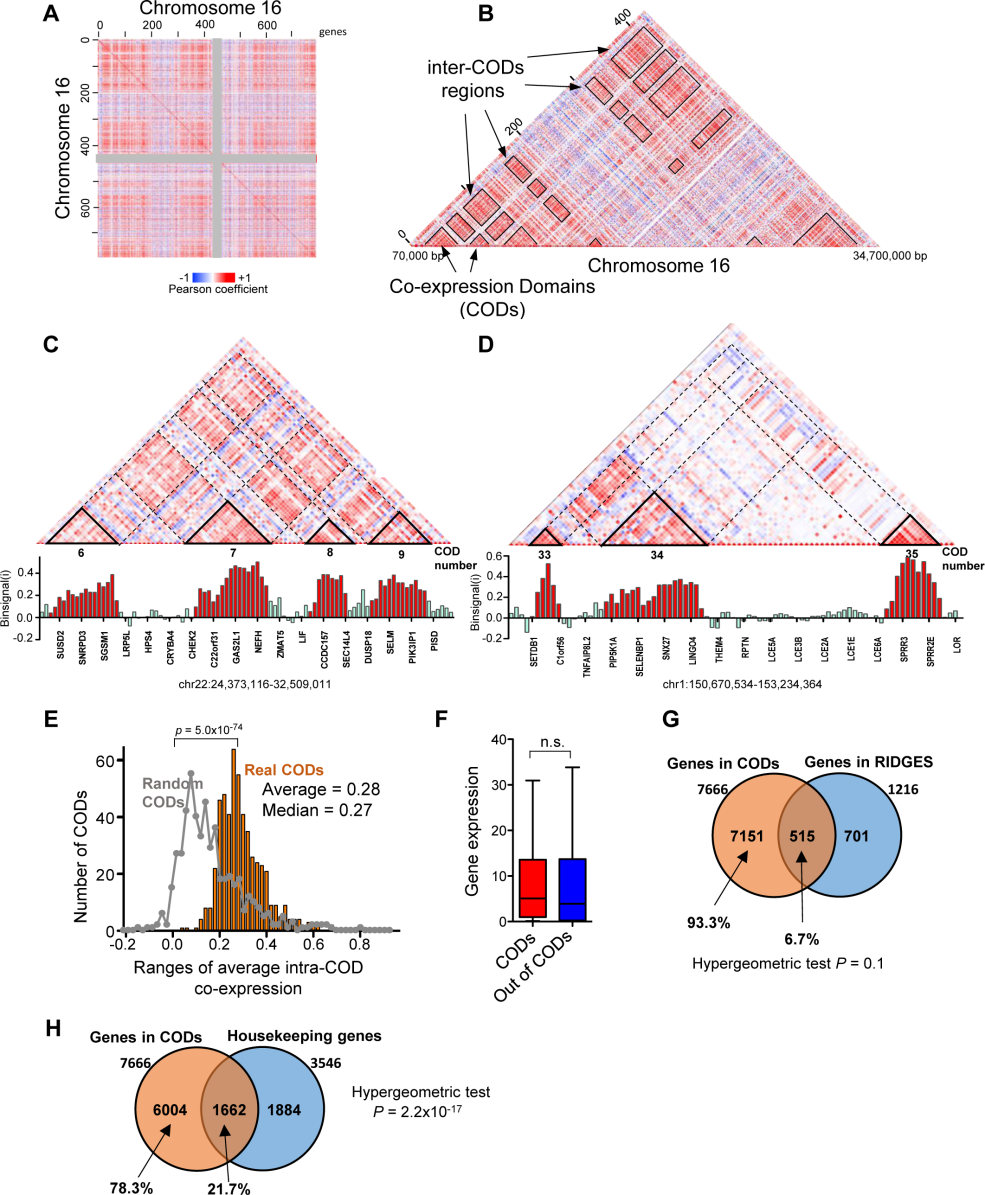
Gene expression is organized into CODs. (A) Heat map representation of the coexpression matrix of chromosome 16. Each pixel represents the Pearson coefficient of the correlation between expressions of gene *i* (columns) and gene *j* (rows) in 100 normal breast tissue samples. Coexpression ranges from –1 (blue) to +1 (red). Genes are arranged in the chromosomal order. The centromeric region is depicted in grey for reference. (B) Detail, seen as triangle, of the heat map shown in (A). Contiguous genes with higher coexpression coefficients between them than with surrounded genes were termed coexpression domains (CODs) and are highlighted in the heat map. Regions of the matrix corresponding to pairs of genes *i*, *j* that reside in different CODs (inter-CODs) are also highlighted. (C, D) Upper panel. Detail of regions of the heat maps of coexpression of chromosome 22 and 1. CODs, determined as defined in Methods, are highlighted and numbered according to S1 Table. Lower panel. Histogram of *binsignal(i)* parameter for each gene *i*. The name of every fifth gene is displayed. Bars of genes located within CODs are in red. Genomic coordinates of the regions in hg19 are provided. (E) Distribution of average intra-COD coexpression values. Distribution obtained with randomized CODs of the same size is also plotted on the grey line. Distributions are significantly different, with p = 5.0 x 10^−74^ (Mann-Whitney test). (F) Gene expression (RNA-seq data, RSEM normalized) of genes that are inside or outside of CODs. Differences were not significant (n.s.; Mann-Whitney test). (G) Venn diagram showing overlap between COD genes and RIDGE genes (S2 Table). (H) Venn diagram showing overlap between COD genes and housekeeping genes.

We obtained RNA-seq expression data of 20,502 genes from 100 normal breast tissue samples from The Cancer Genome Atlas (TCGA) (see Methods). Genes were divided into 23 groups according to its chromosome location. Twenty-three correlation matrices, one for each chromosome, were then constructed in which the matrix entry m_ij_ was the Pearson correlation coefficient between the expressions of gene *i* (in the *i*-th row) and *j* (in the *j*-th column) in the 100 samples. Coexpression between the genes of each chromosome was visualized in heat maps, whereby genes are ordered in the 5′–3′ chromosomal order and color intensities indicate correlation coefficient. The heat maps showed regions of positive and negative correlations and, in some chromosomes, a very sharp plaid pattern (Fig 1A for chromosome 16, see S1 Fig for all chromosomes). Close analysis of the diagonals of the maps revealed regions containing highly coexpressed collinear genes. We called these regions Coexpression Domains (CODs) (Fig 1B). Physical gene maps of two CODs are shown in S2 Fig.

To systematically identify all CODs in the genome, we adapted a method recently designed for TAD identification, TopDom [44]. Following their method, first we calculated an average coexpression value between a window of four genes upstream and downstream around a gene *i* (*binsignal*(i)). As shown in Fig 1C and 1D, the value of *binsignal(i)* is relatively high inside CODs but low between CODs. COD boundaries were determined as regions where the *binsignal(i)* value changes significantly (p < 0.05). CODs were defined as regions with a high *binsignal(i)* value (above average *binsignal(i)* of the genome) delimited by statistically significant boundaries (see Methods). CODs with less than four genes were discarded. Randomization of the positions of the genes dramatically altered the distribution of the *binsignal(i)* values, such that CODs could not be identified (S3 Fig). Using this system, we identified 524 CODs in the human genome that were distributed proportionally to the chromosome gene number (S4A and S4B Fig). Of the 20,502 genes analyzed, 7666 (37.4%) were found within CODs, with an average of approximately 14.6 genes per COD and a median of 10 genes per COD (S5A Fig). This distribution was remarkably similar in most chromosomes (S4C Fig). Median length was around 0.9 Mb per COD (S5B Fig). We then compute the average intra-COD coexpression as the average of Pearson coefficients of coexpression among all pairs of genes within the COD. Average intra-COD coexpression values of real CODs were significantly higher than average intra-COD coexpression of randomized CODs of the same size and separated by the same distance as real CODs (p = 5.0x10^-74^, Bonferroni-corrected Mann-Whitney test) (Fig 1E). S1 Table lists all identified CODs, their gene composition, the average intra-COD coexpression value, and the p-value of the boundaries.

We next tested whether CODs correlate to previously-described gene clusters. Caron et al. described clusters of highly expressed genes (called RIDGEs, Regions of Increased Gene Expression) in the human genome [19]. COD genes did not display higher levels of expression than non-COD genes (Fig 1F), indicating that CODs are not exclusively formed by highly expressed genes. Furthermore, COD genes were not enriched in genes located in RIDGEs (S2 Table) (Fig 1G), indicating that CODs do not correspond to RIDGEs. Further, Lercher et al. reported clustering of housekeeping genes on the human genome [18]. We investigated the presence of housekeeping genes in CODs. Only 21.7% (1662) of the 7666 genes found in CODs were housekeeping genes, as defined by Eisenberg and Levanon [45] (Fig 1H). While these data indicate a significant enrichment of housekeeping genes in CODs (hypergeometric test p = 2.2 × 10^−17^), they also imply that most CODs genes (78.3%) are not housekeeping genes.

Tandem duplicated genes are often coexpressed because they use to have similar promoters [2, 46]. Of the 148 gene clusters of more than 4 genes previously defined in the human genome [47], 65 were found in CODs. For example, COD 36 of chromosome 1 is formed by 13 members of the *S100A* gene family; CODs 3 and 5 of chromosome 6 contain 29 and 15 canonical histone coding genes, respectively; and COD 16 of chromosome 5 contains protocadherin alpha and beta clusters, among other genes (S1 Table).

We performed gene ontology (GO) analysis of all genes included in CODs. Interestingly, COD genes were slightly but significantly enriched in categories involved in DNA and RNA metabolism, such as nucleic acid metabolic processes (Bonferroni-corrected hypergeometric test p = 1.7 × 10^−9^), gene expression (p = 7.06 × 10^−8^), and RNA biosynthetic processes (p = 7.09 × 10^−8^); this is consistent with the slight enrichment in housekeeping genes. As expected, random COD genes were not enriched in any GO category.

**Fig 2:**
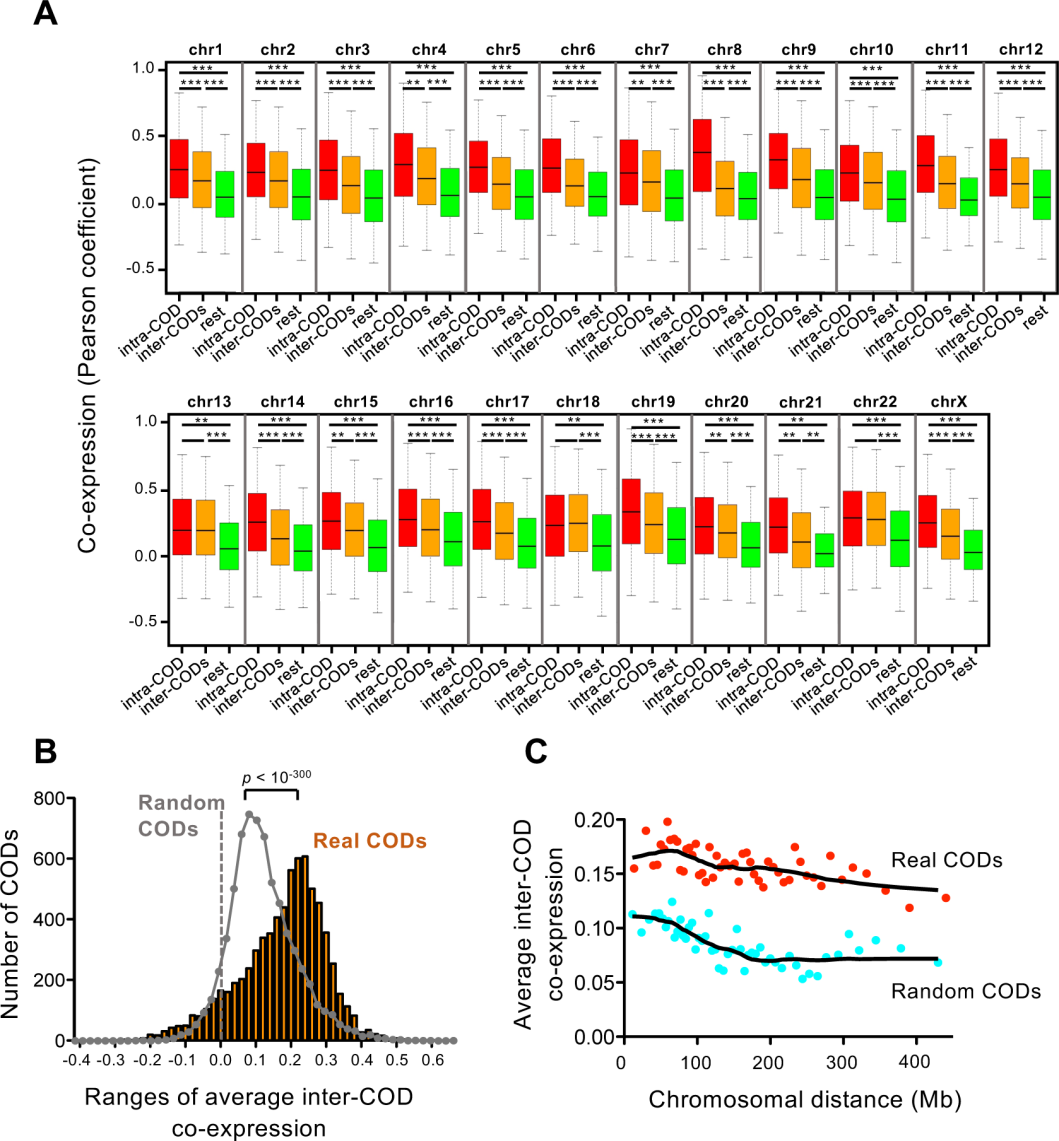
Characterization of inter-COD coexpression. (A) Box plot of coexpression (Pearson correlation coefficient) of pair of genes that reside in the same COD (intra-COD), in two different CODs (inter-COD), and rest of pairwise gene coexpression (rest). *** p < 10^−100^; ** p < 10^−10^, Bonferroni-corrected p-values of the Mann-Whitney test. (B) Distribution of average inter-COD coexpression values. Distribution obtained with randomized CODs of the same size is also plotted on the grey line. Distributions are significantly different with p < 10^−300^ (Bonferroni-corrected Mann-Whitney test). (C) Dependence of distance between CODs of the average inter-COD coexpression. Average inter-COD coexpression data were ordered according to inter-COD distance and binned into 50 groups. Average distance and average inter-COD coexpression of each group are represented. Red dots, real CODs; blue dots, randomized CODs. Data from real CODs were significantly different respect to data from random CODs, with p < 0.01 for all bins (Mann-Whitney test).

Interestingly, we also observed that genes located in CODs are often coexpressed with other genes also located in CODs, creating the plaid pattern in the heat maps (Fig 1A, 1B, 1C and 1D; S1 Fig). Coexpressions between pairs of genes located in the same COD (intra-COD) are significantly higher than coexpressions between pairs of genes located in different CODs (inter-COD), and both of these are higher than the rest of pairwise gene coexpressions (Fig 2A). Randomization of the CODs dramatically decreased these differences (S6 Fig). We then defined an average inter-COD coexpression between two CODs (containing *i* and *j* genes, respectively) as the average of Pearson coefficients of coexpression among all the *i*–*j* pairs of genes. The distribution of average inter-COD coexpressions from real CODs was very significantly different from the values obtained using randomized CODs of the same size (p < 10^−300^, Bonferroni-corrected Mann-Whitney test) (Fig 2B). We selected an |average Inter-CODs coexpression| ≥ 0.2 as strongly significant for future calculations. Thus, 44,85% of the possible intra-chromosomal pairs of CODs are positively co-regulated (average inter-CODs coexpression ≥ 0.2), and 0.53% are negatively co-regulated (average inter-CODs coexpression ≤ –0.2). Examples of positive coexpression, no coexpression, or negative coexpression inter-CODs are shown in Fig 1C and 1D, and S7 Fig, respectively. These data indicate the existence of mechanisms of co-regulation of gene expression that operate over long distances. We also observed that the average inter-COD coexpression is maintained along very large distances, and even between CODs placed in different arms of the same chromosome. In fact, inter-COD coexpression was higher than expected by chance for all distances (Fig 2C, Mann-Whitney test p < 0.01 for all bins). In summary, our data suggest existence of a level of co-regulation inter-CODs along the chromosomes.

### CODs in breast cancer cells

**Fig 3:**
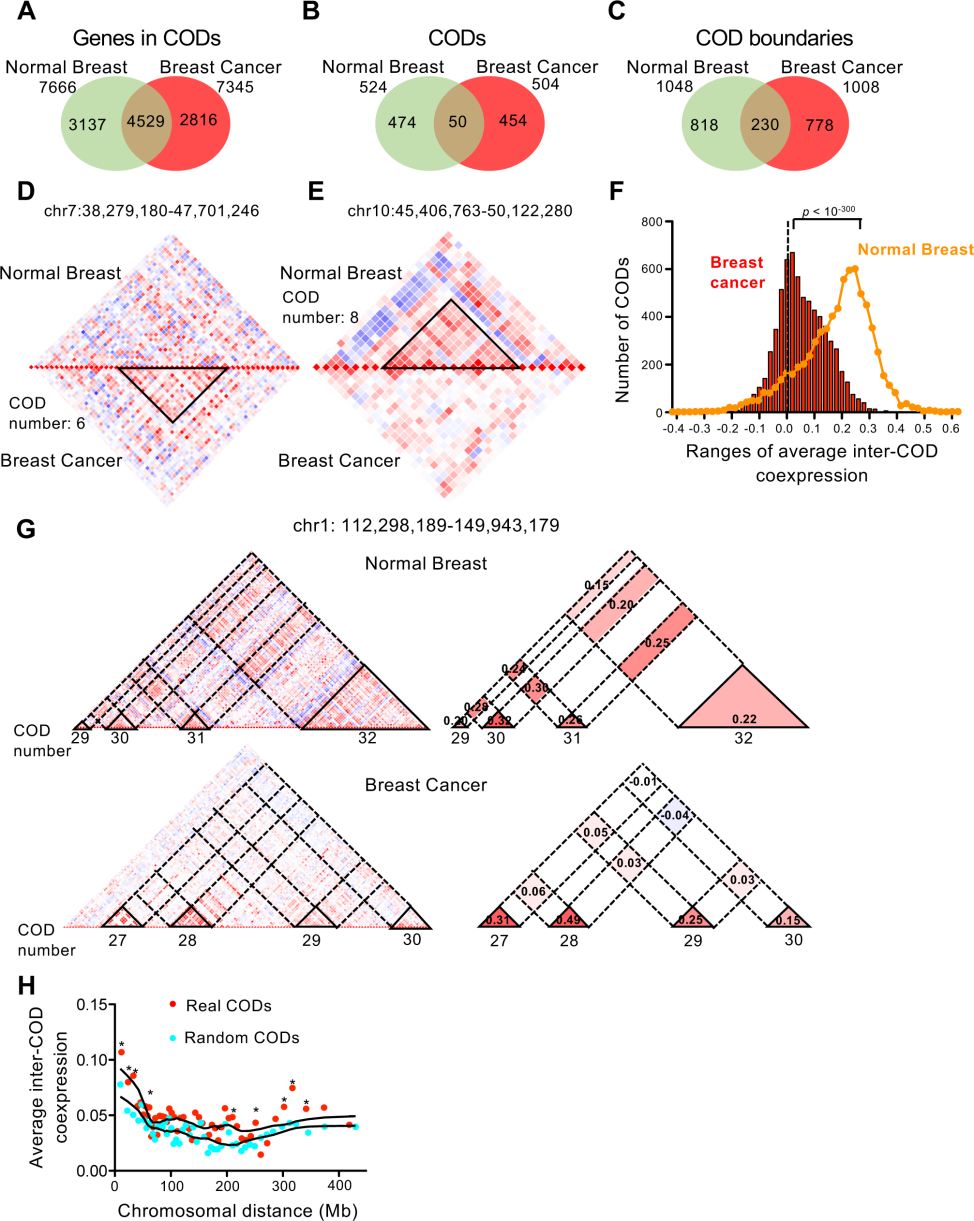
CODs in breast cancer cells. (A–C) Venn diagrams showing the number of overlapping genes in CODs (A), overlapping CODs (at least 80% of COD genes coincidence) (B), and COD boundaries (C), between normal breast samples and breast cancer samples. (D) Example of COD present in breast cancer but not in normal breast tissue. (E) Example of COD present in normal breast tissue but not in breast cancer tissue. (F) Distribution of average inter-COD coexpression values in breast cancer. Line shows same parameter in normal breast tissue for comparison. Both distributions are significantly different, with p < 10^−300^ (Bonferroni-corrected Mann-Whitney test). (G) Detail of heat maps of coexpression of a region of chromosome 1 in normal (upper panels) and cancer (lower panels) breast tissue. Right panels, schematics highlighting intra-CODs and inter-CODs regions. Average inter-COD and intra-COD coexpression values are provided. (H) Dependence of distance between CODs of the average inter-COD coexpression in breast cancer. Average inter-COD coexpression data were ordered according to inter-COD distance and binned into 50 groups. Average distance and average inter-COD coexpression of each group were represented. Red dots, real CODs; blue dots, randomized CODs. Significantly different data points (p < 0.01, Mann-Whitney test) between real CODs and random CODs are indicated with asterisks (*).

We next compare the previously identified CODs from normal breast samples with CODs identified in breast cancer samples. For that, we collected RNA-seq expression data of 20,502 genes from 369 breast cancer tumors from TCGA (see Methods) and used it to construct correlation matrices encompassing all pairs of genes of each chromosome. After applying our COD identification method, we obtained 504 CODs in the breast cancer genome (S1 Table), which is similar to the 524 found in normal tissue. Likewise, the number of CODs per chromosome (S4D and S4E Fig), and the number of genes per COD (S4F and S5C Figs), were also similar between the breast cancer samples and normal samples. Furthermore, distribution of average intra-COD coexpression values from cancer samples was not significantly different from the normal tissue distribution (Bonferroni-corrected Mann-Whitney test > 0.01) (S5E Fig). These data suggest that gene expression is also organized in CODs in the tumor genome. We then compared the overlap between normal and cancer CODs. We found that 59.1% of the genes found in CODs in normal breast were also found in CODs in breast cancer genomes (Fig 3A). In sharp contrast, only 9.5% of the CODs overlapped in more than 80% of the genes (Fig 3B), indicating a strong reorganization of CODs from the normal to the cancer state. Interestingly, about 22.5% of the COD boundaries coincided between normal and cancer samples (Fig 3C), which is significantly higher than the coincidence obtained between random normal CODs and real cancer CODs (14.6%) (p < 0.0001, χ^2^ test). These data suggest that often breast cancer CODs results from fusion and division of normal CODs or by shifting of one of the borders but not the other.

Some of the cancer-specific CODs are related to known breast cancer amplified regions. For example, CODs 5, 6 and 7 of chromosome 8, COD 13 and 14 of chromosome 11, COD 15 of chromosome 17, and COD 6 of chromosome 20 are absent in normal breast tissue and correspond to the well-known breast cancer–associated amplicons in 8p11-p12, 11q13, 17q12, and 20q13 (S8 Fig) ([48–50] and data obtained from TCGA).

Other regions with strong reorganization of CODs between normal and breast cancer samples are not associated to genomic reorganizations. For example, the cancer-specific COD 6 of chromosome 7 (Fig 3D) encompasses 21 genes, of which 10 have been implicated in cancer. Particularly interesting are the genes *PGAM2* [51], *GCK* [52], and *OGDH* [53], which encode enzymes of the glycolysis and the tricarboxylic acid cycle, as well as *POLM [54]* and *POLD2* [55], which encode DNA polymerase μ and a subunit of DNA polymerase δ, respectively, suggesting a possible co-regulation of energetic metabolism and DNA replication genes in breast cancer. Fading of normal breast CODs in cancer was also observed (Fig 3E).

We next compared average inter-CODs coexpression. Strikingly, we observed a very significant decrease of inter-CODs coexpression in breast cancer with respect to normal breast samples (Bonferroni-corrected Mann-Whitney test p < 10^−300^) (Fig 3F and 3G). Using the same criterion that we used for normal tissue (|average inter-CODs coexpression| ≥ 0.2), only 5.1% (283) positive, and 0.06% (4) negative, average inter-COD coexpression values were found. Loss of inter-COD coexpression of a region of chromosome 1 is shown in Fig 3G. In clear contrast to normal tissue, average inter-CODs coexpression decreased very significantly with chromosomal distance in breast cancer samples (Fig 3H). We also observed strong changes of long-range coexpression patterns associated with genomic reorganizations. Thus, the heat map of coexpression of chromosome 8 in cancer changes drastically with respect to the normal breast tissue, probably due to the frequent amplifications of the q arm of this chromosome (S8 Fig). Taken together, these data suggest that long-distance gene co-regulation is impaired in the cancer genome.

We next investigated whether specific CODs can be associated with specific clinicopathological tumor characteristics. For that, we divided the 369 breast tumor collection into two subtypes, according to the presence or absence of metastasis in lymph node of the patients (N0, non-metastatic, versus N1-3, metastatic nodes). The group of patients with metastasis in nodes presented a poor prognosis respect to the group of patients without invaded nodes (Log rack test p-value = 0.0048) (S9A Fig). Expression data of these two sets of tumors were used to determine CODs. We found about 34% (182) of CODs coincidence between N1-3 and N0 tumors (S9B Fig). Among the non-coincident CODs, divisions, fusions, limits shifting and completely different CODs were found. S9C Fig shows an example of a N1-3-specific highly coexpressed COD containing nine genes: *VPS26A*, *SUPV3L1*, *HKDC1*, *HK1*, *TACR2*, *TSPAN15*, *NEUROG3*, *C10ORF35*, *COL13A1* that is not present neither in the N0 tumors nor in the normal breast. High expression of some of these genes has been previously linked to metastasis and poor prognosis [56–58]. The coexpression of these nine genes in tumors with metastatic nodes suggests the existence of mechanisms specific for this type of tumors that coordinate expression of all the genes of this COD.

### Relationship between CODs and TADs

**Fig 4:**
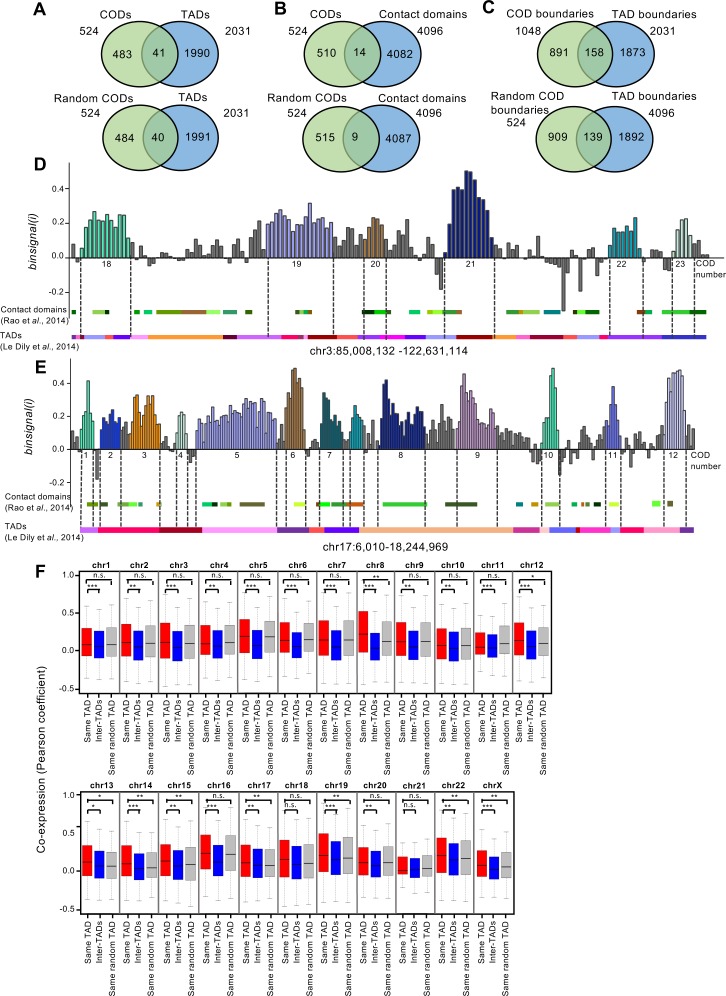
CODs do not correspond to TADs. (A, B) Venn diagrams showing number of real CODs (upper panels) and randomized CODs (lower panels) that match (by at least 80% of the COD length) with TADs in the T47D cell line, as defined by [43] (A), and with contact domains of HMEC cells as defined by [37] (B). (C) Venn diagrams showing overlapping of real COD boundaries (upper panels) and randomized COD boundaries (lower panels) with TAD boundaries. (D, E) *Binsignal(i)* profiles of regions from chromosomes 3 (D) and 17 (E). Genes are arranged in the chromosomal order. Different CODs are depicted in different colors, and numbers correspond to the list in S1 Table. TADs from T47D (as defined by [43]) and contact domains of HMEC cells (as defined by [37]) are represented below each profile. (F) Box plot showing coexpression (Pearson correlation coefficient) of pairs of genes located within the same TAD, in different TADs (inter-TAD), or within randomized TADs of the same size. Bonferroni-corrected p-values from the Mann-Whitney test are provided.

Recent observations suggest that TADs represent fundamental features of chromatin organization [23, 34]. Furthermore, two studies have shown that in some cases, genes lying within the same TAD are co-regulated [41, 43], although other studies have found no correlation between TADs and gene expression [59]. Therefore, we investigated whether CODs correspond to TADs. For this, we used two published datasets of breast cell lines corresponding to two different levels of resolution for TAD analysis: TADs of a median size of about 1 Mb were obtained from the mammary epithelial T47D cell line [43], and high-resolution contact domains of a median size of about 185 kb were obtained from human mammary epithelial cells (HMEC) [37]. First, we calculated the coincidence between CODs and TADs. Only 7.8% and 2.6% of the CODs match (coincidence in at least 80% of the length) with TADs or with contact domains, respectively (Fig 4A and 4B, upper panels). These values were similar (7.6% and 1.7%, respectively) to the coincidence between randomized CODs of the same size and TADs or contact domains (Fig 4A and 4B, lower panels). Likewise, no significant coincidence higher than random was observed when TAD and COD boundaries were compared (Fig 4C). Examples of comparison of CODs with TADs and contact domains distributions are shown in Fig 4D and 4E. In summary, our data demonstrate that CODs do not correspond to TADs or contact domains.

In order to clarify whether TADs are, to some extent, related to co-regulation of gene expression, we compared coexpression between gene pairs positioned in the same TAD (intra-TAD) with coexpression of gene pairs placed in different TADs (inter-TADs) (Fig 4F). Intra-TAD coexpression was higher than inter-TAD coexpression. Similar results were obtained with randomized TADs of the same size, probably due to the fact that linked genes tend to be co-regulated [1, 2, 60, 61]. However, in the chromosomes 8, 12, 13, 14, 15, 17, 19, 22, and X, TAD randomization significantly decreased coexpression with respect to real TADs, indicating that, at least in some chromosomes, gene pairs localized in the same TAD tend to be coexpressed more often than other randomly-selected pairs of close genes. In summary, our data suggest that while TADs do not correspond to the main level of organization of coexpression, they might play a role in gene co-regulation.

### Similar profiles of chromatin contacts in coexpressed genes

After establishing that CODs do not correspond to TADs, we investigated the relationship between coexpression data of normal breast cells and physical chromatin contacts. For this, we used intra-chromosomal Hi-C data at 100 kb resolution from HMEC cells [37]. Since our coexpression matrix is gene-based, and the Hi-C matrices contain pairwise contact frequencies between 100 kb genomic segments, we first assigned connectivity values to each gene pair based on the Hi-C interactions between the corresponding segments in which the genes reside. The frequency of contacts is strongly influenced by sequence proximity and is not a good parameter to be correlated with coexpression of genes situated at very different distances. Lieberman-Aiden et al. defined the correlation interaction profile of a pair of loci as the correlation between distance-normalized contact frequencies of these two loci with the rest of the loci of the chromosome [35]. They assumed that if two loci are close in the 3D volume of the nucleus, they would have highly correlated interaction profiles. We thus compared interaction profiles with gene coexpression. Interestingly, we found that the similarity of interaction profiles of two genes increases with their coexpression in most of the chromosomes (S10 Fig), suggesting that coexpressed genes display similar chromatin contacts. Positive and negative correlation of interaction profiles have been associated to two different compartments—termed A and B—that have different chromatin characteristics [35], which have been recently subdivided into three [62] or five [37] compartments. A detailed analysis of the correlation plots shown in S10 Fig revealed the existence of two or three different linear behaviors in some chromosomes, depending on the range of interaction profile values. The sharp changes of correlation line slope around 0 and 0.2 (see for example chromosomes 7, 12, 16, 19, and 20) suggest that the relationship between coexpression and chromatin contacts is different in different compartments.

**Fig 5:**
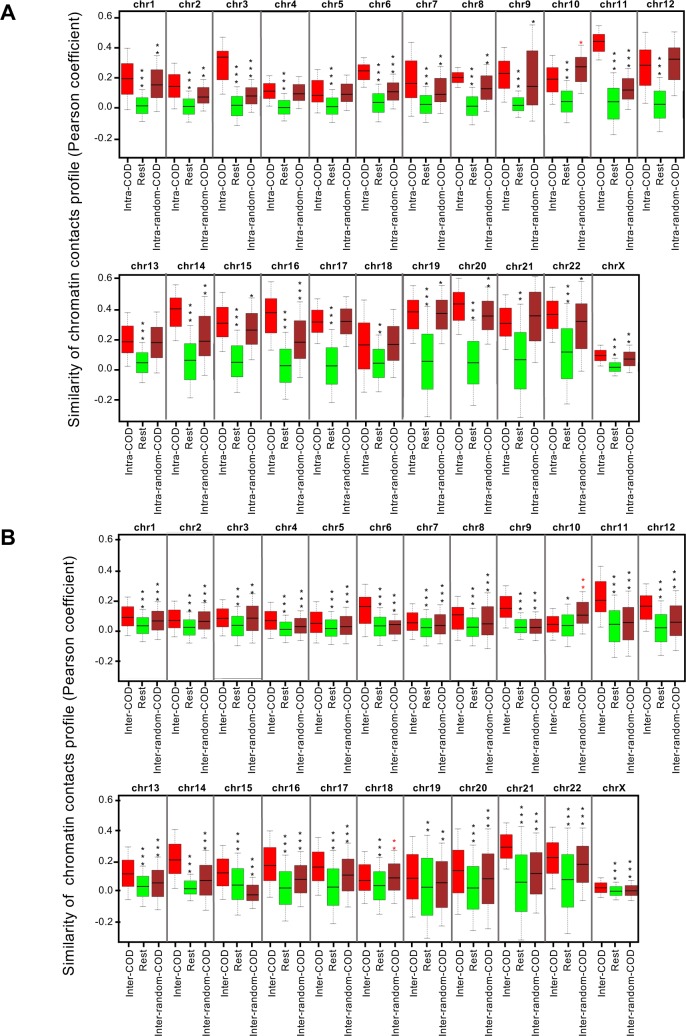
Similarity of chromatin contact profiles of genes in the same COD or in coexpressed CODs. (A) Box plot showing similarity of contact profiles (Pearson correlation of contact frequencies) of gene pairs located in the same COD (intra-COD), rest of gene pairs (Rest), and gene pairs located in the same randomized COD (intra-random-COD). (B) Box plot showing similarity of contact profiles of pairs of genes located in different coexpressed CODs (inter-COD), rest of gene pairs (Rest), and pairs of genes located in different randomized CODs (inter-random-COD). ***p < 10^−100^; **p < 10^−10^; *p < 10^−5^, Bonferroni-corrected p-values of the Mann-Whitney test.

We then analyzed the relationship between interaction profiles and CODs. We found that the profile of contacts between gene pairs in the same COD was more similar than the profile of contacts for the rest of gene pairs (Fig 5A). Randomization of CODs significantly decreased the similarity of the contact profiles in 2/3 of the genome (15 chromosomes) being the effect not significant for chromosomes 4, 5, 10, 12, 13, 17, 18, and 21. These data suggest that in most of the cases, genes within the same COD have similar contact profiles. Next, we analyzed the relationship between the coexpression of genes from different CODs and the pattern of chromatin contacts. We found that the pattern of contacts between two genes placed in different coexpressed CODs (with significant average inter-COD coexpression) is significantly more similar than the contact profile of other pairs of genes. This effect decreased upon randomization of CODs (and therefore, inter-CODs) for all chromosomes except chromosome 10 and the low gene density chromosome 18 (Fig 5B). These data indicate that two genes located in distant coexpressed CODs display similar physical contacts.

## Discussion

### Organization of genome expression in CODs

Clustering of co-expressed human genes was reported several years ago (reviewed in [1, 2]). Different strategies have been used since then to define these clusters, with the expression data from different tissue and from cancerous samples often pooled [16, 18, 19]. This pooling drastically reduces the number and size of statistically significant clusters [63] and creates large discrepancies between studies regarding the size and location of these clusters (see for example [18] and [63]). Here we have used RNA-seq expression data from one single normal tissue (breast) or from one single type of tumor (breast tumor) to determine coexpression domains (CODs) by using a method based in TopDom [44], a software initially designed for TAD identification. We found a very significant organization of the genome into CODs of about 0.9 Mb in size, with a median of about 10 genes per COD. In contrast to other studies [14, 16, 18, 19], we did not consider clusters of less than 4 genes, which rules out bidirectional promoters and deficient termination as possible mechanisms behind COD coexpression. In contrast to the RIDGE domains defined by Caron et al. [19], COD genes are not expressed at higher level than non-CODs genes. Further, while CODs are enriched for housekeeping genes, most COD genes are not housekeeping, differentiating our CODs from the housekeeping gene clusters defined by Lercher et al. [18]. We compared normal breast tissue CODs with breast cancer CODs. As expected, due to the very different pattern of gene expression, the gene composition and the distribution of CODs changes drastically in breast cancer with respect to normal breast tissue, suggesting that CODs are tissue-specific. We also noticed that cancer CODs are strongly influenced by copy number amplification and deletions typically observed in cancer samples, which may also hinder COD identification when normal and cancer samples are mixed. Interestingly, we also found that COD genes tend to be coexpressed with other COD genes of the same chromosome, suggesting a long-range intra-chromosomal co-regulation of CODs.

Which mechanisms are responsible for intra-COD coexpression? One possibility is that genes inside CODs are regulated by cis-regulatory elements, such as enhancers, that affect most or all of the genes of the COD. Enhancers are typically placed in the vicinity of their regulated genes, although some enhancers may act at Mb-scale distances [64, 65]. How many genes can be regulated by one enhancer, and how far away an enhancer can be to still function, are interesting questions that remain to be clarified [64]. Specific for our study is the question of whether about ten genes within a COD can be controlled by the same enhancer (within the same COD). Two recent studies support a highly dynamic interaction between an enhancer and two different promoters [66, 67], suggesting the existence of multi-target enhancers. Much less clear is how to explain inter-CODs coexpression. In other words—how can different CODs separated by dozens of Mb be coordinately regulated? One possibility is that a number of enhancers of different regions (different CODs) cooperate to coordinately control the expression of distant CODs of the genome, as 3D super-enhancers. In fact, spatial enhancer clustering has been reported [68]. This coordinated enhancer function may result in a transient and local high concentration of RNA polymerases and associated factors around a region of the genome, which would coordinately regulate transcription of large sets of genes from different CODs. This hypothesis is consistent with the transcription factories concept developed by Cook and others [69–71]. Interestingly, “specialized” factories in which tissue-specific and/or pathway-specific co-regulated genes are transcribed have been reported, supporting a role of factories in coexpression [33, 72].

### Coexpression and chromatin contacts

The mechanisms previously discussed imply the existence of short- and long-range contacts between different regions of the chromatin. Hi-C studies have shown that chromatin is segmented into self-interacting domains called topological-associated domains (TADs), with median sizes ranging from 185 kb to one Mb, depending on the resolution of the study [37, 40–42]. It is accepted that enhancer activity is limited to the genes that fall within the same TAD [64], and contacts between different TADs have been reported [37]. Therefore, one logical initial possibility was that CODs correspond to TADs. However, our results indicate that CODs do not match with TADs in either normal or cancer breast cell genomes. This is in agreement with the fact that most TADs are invariant between cell types or between cancer and normal tissue, in clear contrast to CODs. To what extend genes inside TADs are co-regulated is unclear. Two studies have shown that under particular conditions (progesterone treatment and genes of X-chromosome involved in differentiation of ESC into epiblast) genes lying within the same TAD are significantly co-regulated [41, 43]. Neems et al., have recently reported TADs enriched for muscle-specific genes [15]. However, most myogenesis-specific genes (87.3%) were located in TADs non-enriched for myogenesis-specific genes. In fact, in many cases genes in the same TAD display different expression patterns [64]. For example, Barutcu et al. did not find gene expression changes in the genes contained in the small number of TADs that change between the non-tumorigenic mammary epithelial MCF-10A and the breast cancer MCF-7 cell lines [59]. Correspondingly, we did not find intra-TAD coexpression higher than random in most of the chromosomes. However, a small, but higher than random, intra-TAD coexpression was observed for chromosomes 8, 12, 13, 14, 15, 17, 19, 22, and X.

Definition of TADs is based on the frequency of chromatin contacts [40]. Consistent with the absence of coincidence between CODs and TADs, genes of the same COD did not display a higher frequency of contacts among themselves than with genes of different CODs. However, we found that genes in the same COD displayed a similar (correlated) profile of contacts in most of the chromosomes. Interestingly, a similar profile of contacts was also found between genes of different but co-regulated CODs. These data are again consistent with the transcription factories model, in which different co-regulated genes can interact with one or a few factories without necessarily having to have interactions between themselves. A recent study using in situ hybridization data from 4,345 genes of the mouse brain reached similar conclusions about the correlation between coexpression and connectivity data [73]. However, that study did not analyze coexpression clusters, maybe due to the small coverage of the genome obtained (with only 4,345 genes). The fact that two loci display a correlated profile of contacts suggests that they are close in space [35]. Indeed, intra-chromosomal coexpression is much higher than inter-chromosomal coexpression [74], which is also consistent with the existence of chromosomal territories [25–27]. Nonetheless, coexpression of genes from different chromosomes also occurs. Whether CODs of different chromosomes can be coexpressed, and whether this also implies that similar inter-chromosomes chromatin contacts occur, remains to be investigated.

Cancer samples showed an overall lower coexpression than normal samples. Since average intra-CODs coexpression was very similar between normal and cancer samples most reduction come from the inter-COD coexpression, suggesting that cancer CODs have, at least partially, lost the capacity of co-regulation with other CODs. Interestingly, the number of transcription factories range from 200 in a primary cells [75] to 2000 in HeLa cells [76]. Although preliminary, due to the small number of cell types analyzed, these data suggest a higher number of factories in cancer cells than in primary cells. If factories are hubs for coexpression, a high number of factories implies less coexpression, which would fit with the lower capacity of coordination between CODs that we observed in cancer cells. Our data are also in agreement with data from Barutcu et al., who have recently reported a decrease in the frequency of inter-chromosomal interactions between small chromosomes, and intra-chromosomal interactions particularly in telomeric and subtelomeric regions of the genome, in breast cancer cells with respect to epithelial non-transformed cells [59].

In summary, our data support that genes are organized into highly coexpressed regions—CODs—that have similar profiles of physical interactions. CODs can also be coexpressed with other CODs, and these also have similar profiles of chromatin contacts. It is tempting to speculate that common physical contacts are the mechanism that determine coexpression. However, whether physical contacts are the cause or consequence of co-regulation requires further investigation.

## Methods

### RNA-seq data and coexpression calculation

Gene expression (RSEM normalized RNA-seq V2 data) of the 20,502 genes available (based on hg19 UCSC Gene standard track (December 2009 version) from 100 normal breast tissue samples and 369 breast tumor samples were collected from TCGA (https://cancergenome.nih.gov/). GDC manifest files for identification of normal and cancer samples used are provided in S1 Appendix and S2 Appendix, respectively.

First, twenty-three different expression vectors–one for each chromosome–were constructed, and genes (gene i (gi)) were sorted according to their 5′ to 3′ chromosomal order, using the assembly hg19 of the human genome.

expr_normal_(gi) = expr gi_sample1_, expr gi_sample2_, … expr gi_sample100_
expr_normal_(gj) = expr gj_sample1_, expr gj_sample2_, … expr gj_sample100_

expr_tumor_(gi) = expr gi_sample1_, expr gi_sample2_, … expr gi_sample369_
expr_tumor_(gj) = expr gj_sample1_, expr gj_sample2_, … expr gj_sample369_

With 1≤ i,j ≤ 20502

expr_normal_ chr n = expr_normal_ | _genes ∈ Chr n_
expr_tumor_ chr n = expr_tumor_ | _genes ∈ Chr n_

With 1≤n≤23, where n is the number of the chromosome.

Then, twenty-three correlation matrices, C, containing the Pearson correlation coefficient between the expression profiles of every pair of genes (gene i (gi), gene j (gj)) was constructed in R (http://www.rproject.org) for each chromosome of each set of data (normal breast and breast tumor), using the *cor* function of the stats package.

C_normal_(i,j) chr n = cor(expr_normal_(gi), expr_normal_(gj)), gi,gj ∈ Chr n
C_tumor_(i,j) chr n = cor(expr_tumor_(gi), expr_tumor_(gj)), gi,gj ∈ Chr n

With 1≤n≤23, where n is the number of the chromosome.

Coexpression matrices heat maps were visualized using Gitools 2.3.1 version [77]. In all heat maps genes are arranged in the chromosomal order. Centromere coordinates were obtained from the USCS Genome Browser through the Table Browser (http://genome.ucsc.edu/cgi-bin/hgTables).

### Detection of CODs

We designed a method to determine coexpression domains (CODs) based on methods used to identify TADs, such as the directionality index [40] and the TopDom methods [44]. The input data are the coexpression matrices of each chromosome, where each position contains Pearson coefficient values between any two genes. For each gene, we computed an average coexpression signal between upstream and downstream regions around its position as previously defined in the TopDom method.

$$binsignal\;(i)=1/w2\overset{w}{\underset{l=1}{\sum }}\overset{w}{\underset{m=1}{\sum }}\mathrm{coexpression}\;\mathrm{value}(U_{i}(l),\;D_{i}(m))$$

where U_i_ = {i-w+1,…i-1, i}, D_i_ = {i+1, i+2,..i+w}, and w is the size of the window around i. As shown in Fig 1C and 1D, this parameter is high for genes around the center of the CODs and decreases at COD boundaries and at chromosomal regions between CODs. We defined CODs as regions of *binsignal(i)* ≥ 0.15 delimited by 5′ and 3′ significant boundaries. We selected 0.15 because it is the average *binsignal(i)* of the genome. Boundaries are defined as regions larger than three genes with *binsignal(i)* < 0.15 that delimit regions with significantly different *binsignal(i)* (p < 0.05). We determine boundaries to be statistical significance by computing a Student´s t-test between the four upstream (i-w+1,… i-2, i-1, i), and the four downstream (i+1, i+2,…, i+w) *binsignal(i)* values for each gene i. Less than two consecutive genes with *binsignal(i)* < 0.15 inside a COD are allowed if these segments are not boundaries. A script for CODs determination (CODfinder) was written in R and deposited in the GitHub repository (https://github.com/joseguem/CODfinder.git).

To determine the best gene window size w, we run the program using different w values for chromosome 1 and calculated the average intra-COD coexpression of the CODs identified. Similar average intra-COD coexpressions were obtained with w between 3 and 6 genes, indicating the robustness of the system of identification of CODs (S11A Fig). We selected w = 4 for the rest of the study because is the window for which average intra-COD coexpressions was maximum. S11B and S11C Fig show variation in the number and size of CODs depending on w value.

For randomization of gene order along chromosome 1 in S3 Fig, gene positions were shuffled using the *sample* function available in R base package.

### COD analysis

Genomic coordinates of CODs, according to human genome assembly hg19, were specified by using the first nucleotide of the first COD gene and the last nucleotide of the last COD gene, irrespective of gene orientation. These coordinates were used to determines COD length in bp. To compare expression of genes in CODs with those outside of CODs, the average of RSEM normalized data of expression of every gene in the 100 normal breast samples was computed. COD genes were compared to a list of housekeeping genes obtained from https://www.tau.ac.il/~elieis/HKG/ [45]. Venn diagrams were constructed using the Venn Diagram Generator (http://www.pangloss.com/seidel/Protocols/venn.cgi). The hypergeometric tests were performed in R using the *dhyper* function from the stats package.

For box plots of Fig 2A, we called intra-CODs coexpressions to the Pearson coexpression coefficients of all pairwise combinations between the genes inside the same COD. We called inter-CODs coexpressions to the Pearson coexpression coefficients of all pairwise combinations between the genes from two different CODs within the same chromosome. Pearson coexpression coefficients of all pairwise combinations between the genes that are not in CODs or between a COD gene and a non-COD gene were considered the rest of the intra-chromosomal coexpressions. These data were extracted from each chromosomal coexpression matrix (C_normal_(i,j)_chr_n or C_tumo_(i,j)_chr_n).

Gene ontology functional categories and pathway enrichment were analyzed using WebGestalt software packages (http://www.webgestalt.org/) [78]. Bonferroni-adjusted p-values of the hypergeometric tests were used to determine enrichment significance.

### Cancer CODs analysis

For comparison of normal and cancer CODs, coincidence of at least 80% of normal COD genes was required. For determination of CODs boundaries coincidence between normal and cancer samples a discrepancy of 10% of the number of normal CODs genes was allowed.

Gene copy number data (relative linear copy number from Affymetrix SNP6) corresponding to the 369 analyzed breast tumor samples were obtained from TCGA through cBioPortal (http://www.cbioportal.org/) [79]. The gene copy number profiles in S8 Fig correspond to the average gene copy number values plotted according to the chromosomal gene order. http://dgd.genouest.org/.

Clinicopathological data (N stage and survival) of patients in the breast tumors cohorts were obtained from TGCA. Cancer population was subdivided into two according to N stage. The N grade indicates whether lymph nodes have metastasis N1, N2, N3) or not (N0), respectively. No subdivisions of N stages were used (e.g., N1a, N1b, and N3 were considered as T1-3). Kaplan-Meier survival plots were constructed using Prism 5 (GraphPad). Significance of the difference between groups was comutes using the Log-rank test.

### Determination of RIDGEs regions

We used our average gene expression data from 100 normal breast samples to determine RIDGEs, according to the method described by Caron et al. [19] with some modifications. For this, genes were ordered according to the chromosomal order. Then, for each gene i the median expression of a moving window of 39 genes (19 upstream and 19 downstream of i) was calculated. We found 45 regions with 10 or more consecutive moving medians higher than twice the genomic median. These regions were considered RIDGEs. Genes and coordinates of identified RIDGEs are listed in S2 Table.

### Comparison between CODs and TADs

Genomic coordinates, according to human genome assembly hg19, of Topologically Associating Domains (TADs) from the cell line T47D [43] were provided by M. Marti-Renom (CRG, Barcelona). Genomic coordinates of contact domains from HMEC cells [37] were collected from the Gene Expression Omnibus (GEO) at NCBI (accession number GSE63525). CODs and TADs with at least 80% coincidence of the length were considered as coincident. To determine COD-TAD coincidence, 5′ and 3′ boundary coordinates of every COD of each chromosome were compared with the 5′ and 3′ boundary coordinates of every TAD of the same chromosome. To estimate boundary overlap, every COD boundary was compared with every TAD boundary (irrespectively of the 5′ or 3′ position), with a discrepancy of less than 10% of the COD length allowed. Contact domains were calculated similarly.

In order to investigate whether gene pairs residing within the same TAD had higher coexpression than pairs of genes residing in different TADs or in non-TADs, genes were first assigned to TADs. For that genomic coordinates of genes obtained from the UCSC Genome Browser through the Table Browser (http://genome.ucsc.edu/cgi-bin/hgTables). Genes were then assigned to TADs of the corresponding chromosomes according to their midpoint coordinate. After that, pairwise coexpression of genes (Pearson coefficient correlation) within the same TAD was compared with that of genes located in different TADs, and the significance of the differences was estimated using the Bonferroni-corrected Mann-Whitney test. To investigate whether the significantly higher coexpression of genes placed in the same TAD is a consequence of spatial proximity, TAD positions were randomized but keeping exactly the same TAD size. For randomization, TAD genomic coordinates were inverted 3′ to 5′ in each chromosome. Similar results were obtained when TAD borders were shifted 100,000 bp upstream or downstream.

### Hi-C data and contact matrix normalization

Intra-chromosomal Hi-C data at 100 kb resolution of HMEC cells were collected from GEO at NCBI (accession number GSE63525) [37]. Contact matrices of each chromosome were normalized using KR-Normalization [80, 81] as described [37]. The O/E (“observed over expected”) matrices, which correct for the increased number of contacts due to sequence proximity, and the Pearson correlation matrices of the O/E, which identifies spatial relationships between loci by looking for correlations in their contact patterns, were constructed as described [35]. Next, genes were assigned to the 100 kb anchors, and connectivity data were assigned to pairs of genes based on the corresponding anchor pairs in which the genes reside. Vectors were then constructed that assign to every possible pair of genes of a chromosome their corresponding coexpression (Pearson correlation between expressions) and connectivity (normalized contacts, or Pearson correlation matrices of the O/E). For S10 Fig, connectivity data (Pearson correlation matrices of the O/E) were ranked and grouped into 20 bins with the same number of elements. The average value of connectivity of each bin was then represented against the average coexpression of the corresponding pairs of genes. For Fig 5, box plots of connectivity values (Pearson correlation matrices of the O/E) of pairs of genes that reside in the same COD (intra-COD) or in different CODs (inter-CODs) were compared with the same parameter as the rest of gene pairs of each chromosome. To estimate the random values in each chromosome, COD borders were randomized as described below.

### Randomization and statistic methods

Robust randomization of the COD borders was performed by inverting the chromosomal coordinates from 5′–3′ to 3′–5′ on each chromosome, similarly as described in [43]. This method allows the COD size, the distance between CODs, the genomic context, and the gene proximity to be maintained while changing the gene composition of the CODs. Similar results were obtained when randomization was performed by shifting the COD borders 100,000 bp upstream or downstream.

Student´s t-test and Mann-Whitney test with confidence interval 95% were computed in R using *t*.*test* and *wilcox*.*test* functions from stats package. To test significance of overlapping in Venn diagrams, the hypergeometric tests were performed in R, using the *dhyper* function from the stats package.

### Accession numbers

Genomic coordinates of contact domains and Intra-chromosomal Hi-C data at 100 kb resolution, from HMEC cells [37] were collected from the Gene Expression Omnibus (GEO) at NCBI (accession number GSE63525).

## Supporting information

S1 Fig Heat map representation of the coexpression matrix of all chromosomes. Each pixel represents the Pearson coefficient of the correlation between expressions of gene *i* (columns) and gene *j* (rows) in 100 normal breast tissue samples. Coexpression ranges from –1 (blue) to +1 (red). Genes are arranged in the chromosomal order. Centromeric region is depicted in grey for reference.
(PDF)

S2 Fig Detail of two coexpression Domains (CODs) and physical gene map. Genes are arranged in the chromosomal order. Gene symbol names are shown. Screenshots of the UCSC Genome Browser of the indicated region of chromosomes 1 and 22 are shown below the heat maps.
(PDF)

S3 Fig Histogram of *binsignal(i)* values of a region of chromosome 1. Numbers in the abscises axis correspond to the chromosomal order of genes (A) or to a randomized order of genes (B). Genomic coordinates, according to human genome assembly hg19, are provided.
(PDF)

S4 Fig Chromosomal distribution of CODs in normal or breast cancer samples. (A, D) Histogram of number of CODs per chromosome in normal breast (A) or breast cancer (D) samples. (B, E) Histogram of density of CODs per chromosome (number of CODs per 100 genes) in normal breast (B) or breast cancer (E) samples. (C, F) Histogram of number of genes per COD in the different chromosomes in normal breast (C) or breast cancer (F) samples.
(PDF)

S5 Fig COD characterization in normal and breast cancer samples. (A, C) Number of genes per COD distribution in normal (A) and cancer (C) breast samples. (B, D) Size distribution of CODs in normal (B) and cancer (D) breast samples. (E). Distribution of average intra-COD coexpressions of breast cancer CODs. Distribution of the same parameter of normal breast tissue is also plotted for comparison (yellow line).
(PDF)

S6 Fig Randomization of CODs decreases intra-COD and inter-COD coexpression. (A, B) COD borders were randomized by inverting the chromosomal coordinates from 5′–3′ to 3′–5′ on each chromosome. (A) Box plot of coexpression (Pearson correlation coefficient) of pairs of genes that reside in the same COD (intra-COD), the rest of pairwise gene coexpression (rest), and pairs of genes that reside in the same randomized CODs (Intra-random-COD). (B) Box-plot of coexpression (Pearson correlation coefficient) of pairs of genes that reside in different CODs (inter-COD), the rest of pairwise gene coexpression (rest), and pairs of genes that reside different randomized CODs (Intra-random-COD). ***p < 10^−100^; **p < 10^−10^; Bonferroni-corrected p-values of the Mann-Whitney test.
(PDF)

S7 Fig Example of inter-COD negative coexpression. Detail of heat map of coexpression of chromosome 2. COD numbers according to S1 Table are provided. Inter-COD regions between CODs 9–24, 10–24, and 12–24 are highlighted, and the average inter-COD coexpression is given.
(PDF)

S8 Fig Effect of copy number variation on the coexpression heat maps. The copy number profile in breast cancer samples of chromosomes 8, 11, 17, and 20 were overlaid onto the breast cancer coexpression heat maps. Each pixel of the heat map represents the Pearson coefficient of the correlation between expressions of gene i (columns) and gene j (rows) in 369 breast cancer samples. Coexpression ranges from –1 (blue) to +1 (red). Average gene copy number values (relative linear copy number from Affymetrix SNP6 from TCGA) of the same 369 breast tumor samples were plotted in the chromosomal gene order. Centromeric region is depicted in grey for reference.
(PDF)

S9 Fig Identification of CODs associated with specific clinicopathological tumor characteristics. (A) Comparison of Kaplan-Meier survival plots of patients with N0 tumors (n = 180) and patients N1-3 tumors (n = 186). Log-rank test p-value is provided. (B) Overlapping of CODs (at least 80% identical) between N0 and N1-3 tumors. C) Heat maps showing detail of a COD of chromosome 10 that is present in N1-3 tumors and absent in N0 tumors and normal breast.
(PDF)

S10 Fig Correlation between coexpression and similarity of chromatin contact profile. Chromatin connectivity data of all possible pairs of gene of each chromosome (Pearson correlation matrices of the O/E, see methods) were ranked and grouped into 20 bins with the same number of elements. The average value of connectivity of each bin was then represented against the average coexpression of the corresponding pairs of genes.
(PDF)

S11 Fig Optimization of window size (w) parameter for CODs determination in chromosome 1 or normal breast samples. A) Average intra-CODs coexpression dependence of the w value. B) Variation of the number of CODs detected with different w values. C) Variation of the COD size (in number of genes) with different w values.
(PDF)

S1 Table List of all CODs from normal breast and breast cancer. (XLSX)

S2 Table List of genes in RIDGEs. (XLSX)

S1 Appendix GDC manifest files for identification of TCGA normal breast samples. (TXT)

S2 Appendix GDC manifest files for identification of TCGA cancer breast samples. (TXT)
