# Supplementary figures and images for: Analysis of the relationship between coexpression domains and chromatin 3D organization
Source: PLoS Comput Biol. 2017 Sep 13;13(9):e1005708. doi: 10.1371/journal.pcbi.1005708 (PMC5612749; doi:10.1371/journal.pcbi.1005708)

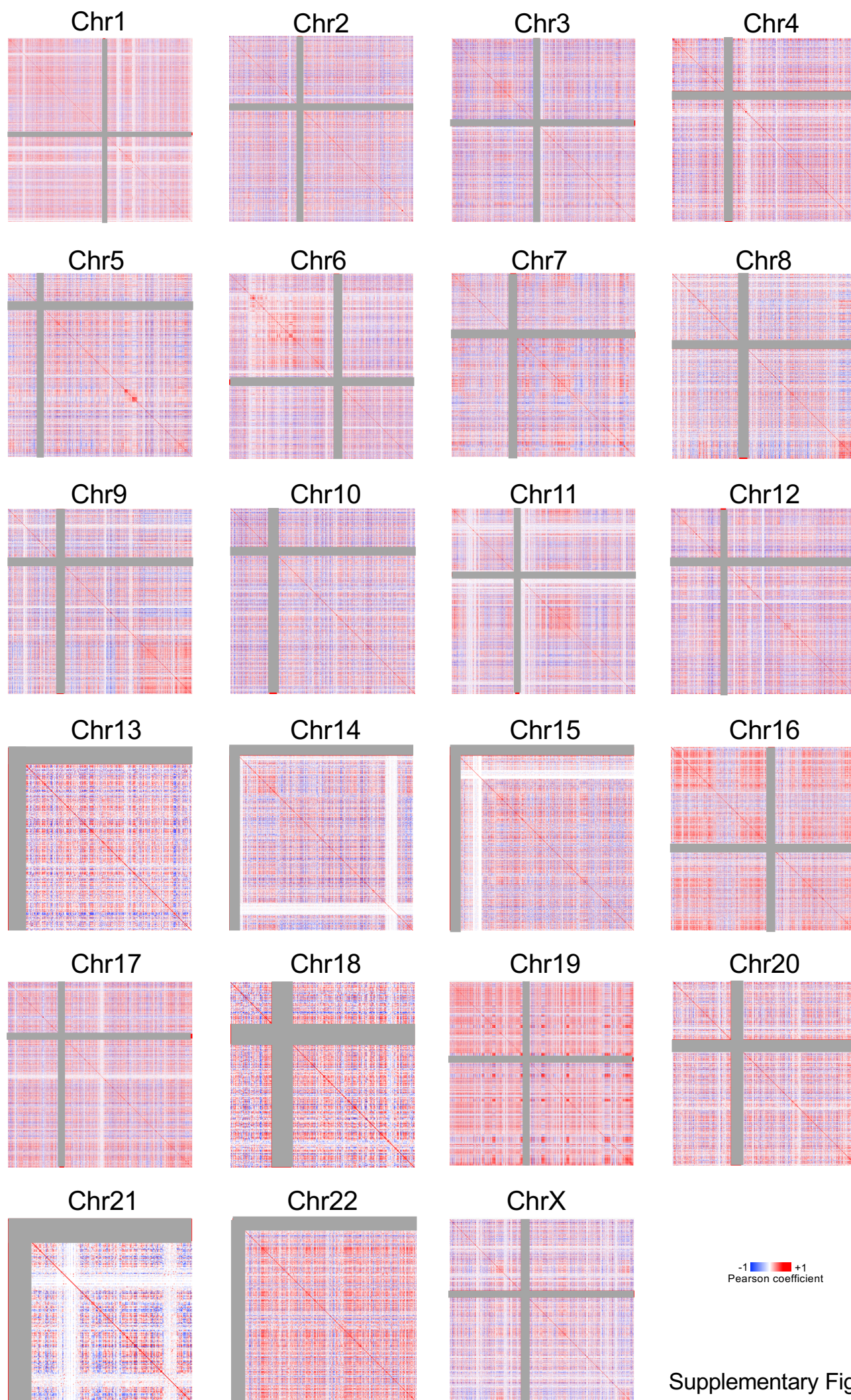

Supplementary Figure S1

Supplement: S1 Fig — Each pixel represents the Pearson coefficient of the correlation between expressions of gene i (columns) and gene j (rows) in 100 normal breast tissue samples. Coexpression ranges from –1 (blue) to +1 (red). Genes are arranged in the chromosomal order. Centromeric region is depicted in grey for reference. (PDF) [file pcbi.1005708.s001.pdf]

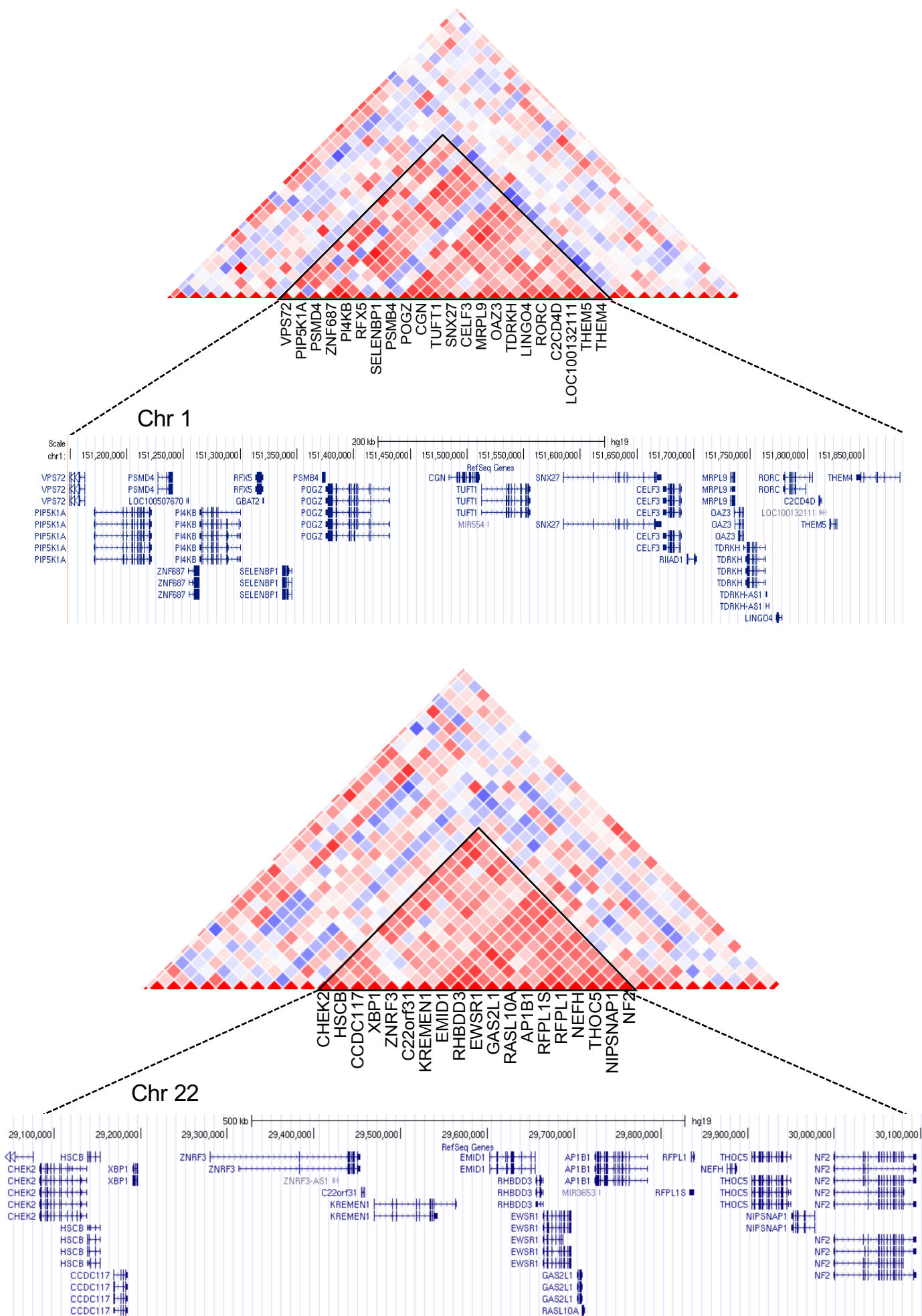

Supplementary Figure S2

Supplement: S2 Fig — Genes are arranged in the chromosomal order. Gene symbol names are shown. Screenshots of the UCSC Genome Browser of the indicated region of chromosomes 1 and 22 are shown below the heat maps. (PDF) [file pcbi.1005708.s002.pdf]

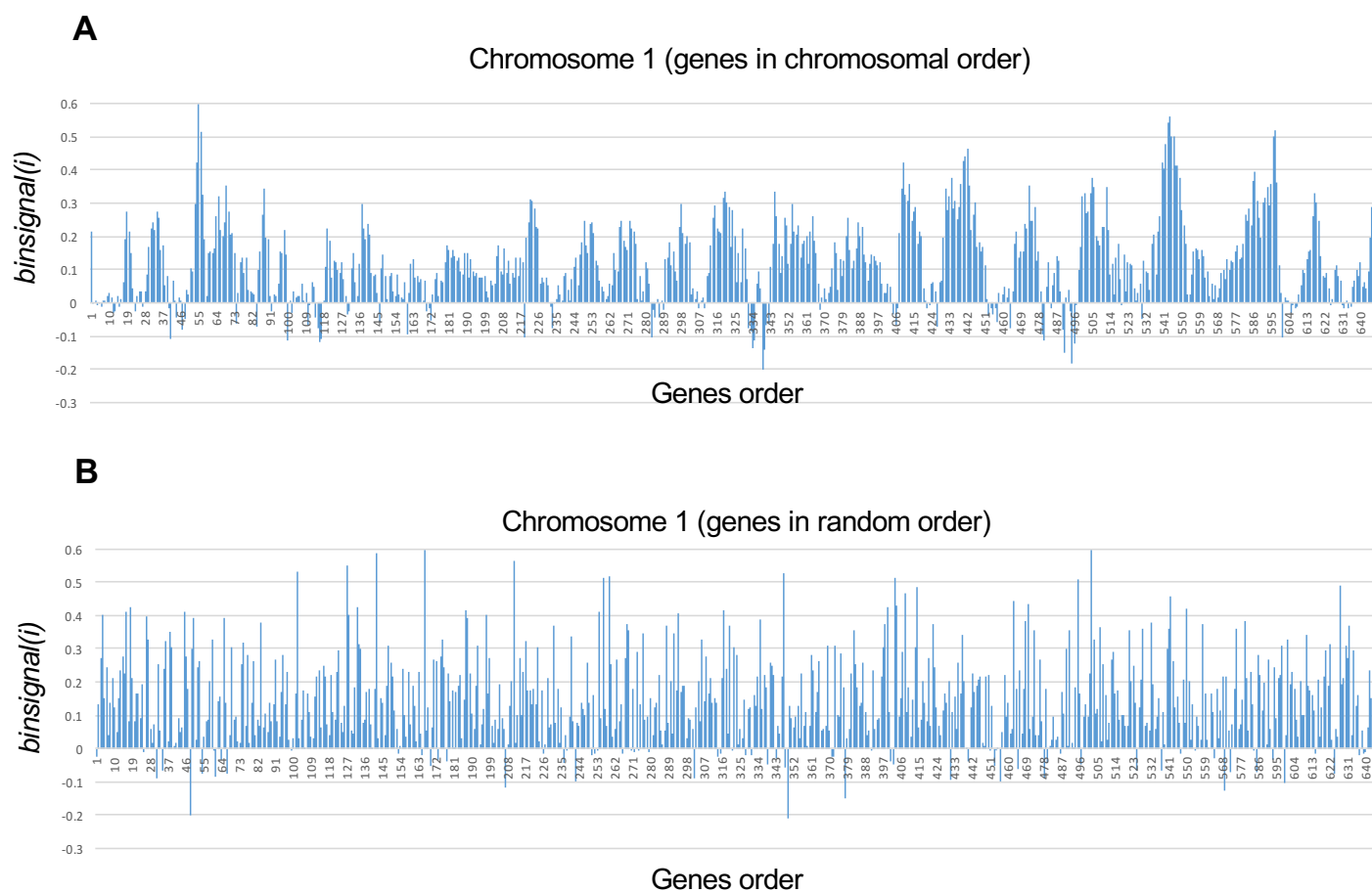

chr1: 158,297,739-249,212,562

Supplement: S3 Fig — Numbers in the abscises axis correspond to the chromosomal order of genes (A) or to a randomized order of genes (B). Genomic coordinates, according to human genome assembly hg19, are provided. (PDF) [file pcbi.1005708.s003.pdf]

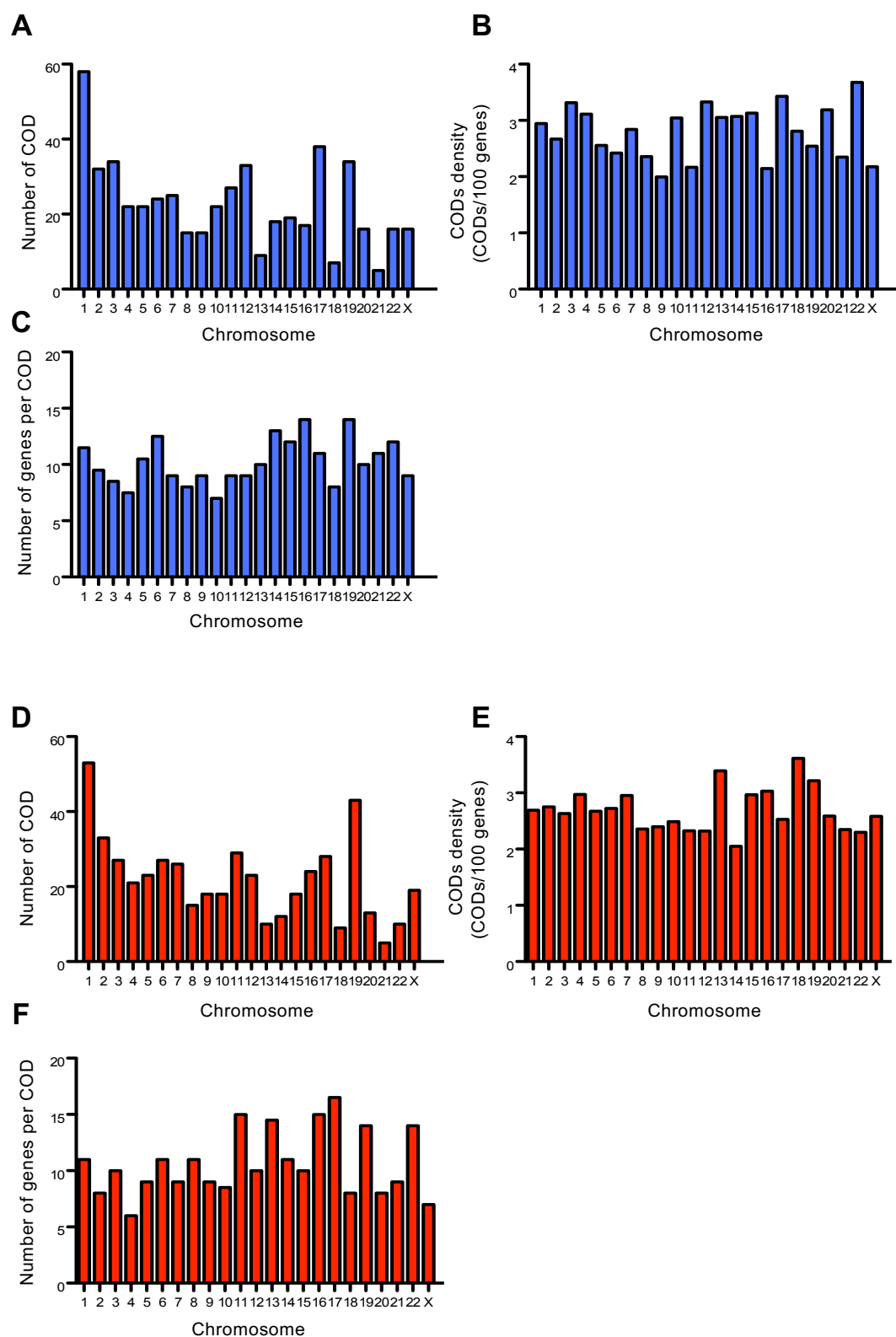

Supplement: S4 Fig — (A, D) Histogram of number of CODs per chromosome in normal breast (A) or breast cancer (D) samples. (B, E) Histogram of density of CODs per chromosome (number of CODs per 100 genes) in normal breast (B) or breast cancer (E) samples. (C, F) Histogram of number of genes per COD in the different chromosomes in normal breast (C) or breast cancer (F) samples. (PDF) [file pcbi.1005708.s004.pdf]

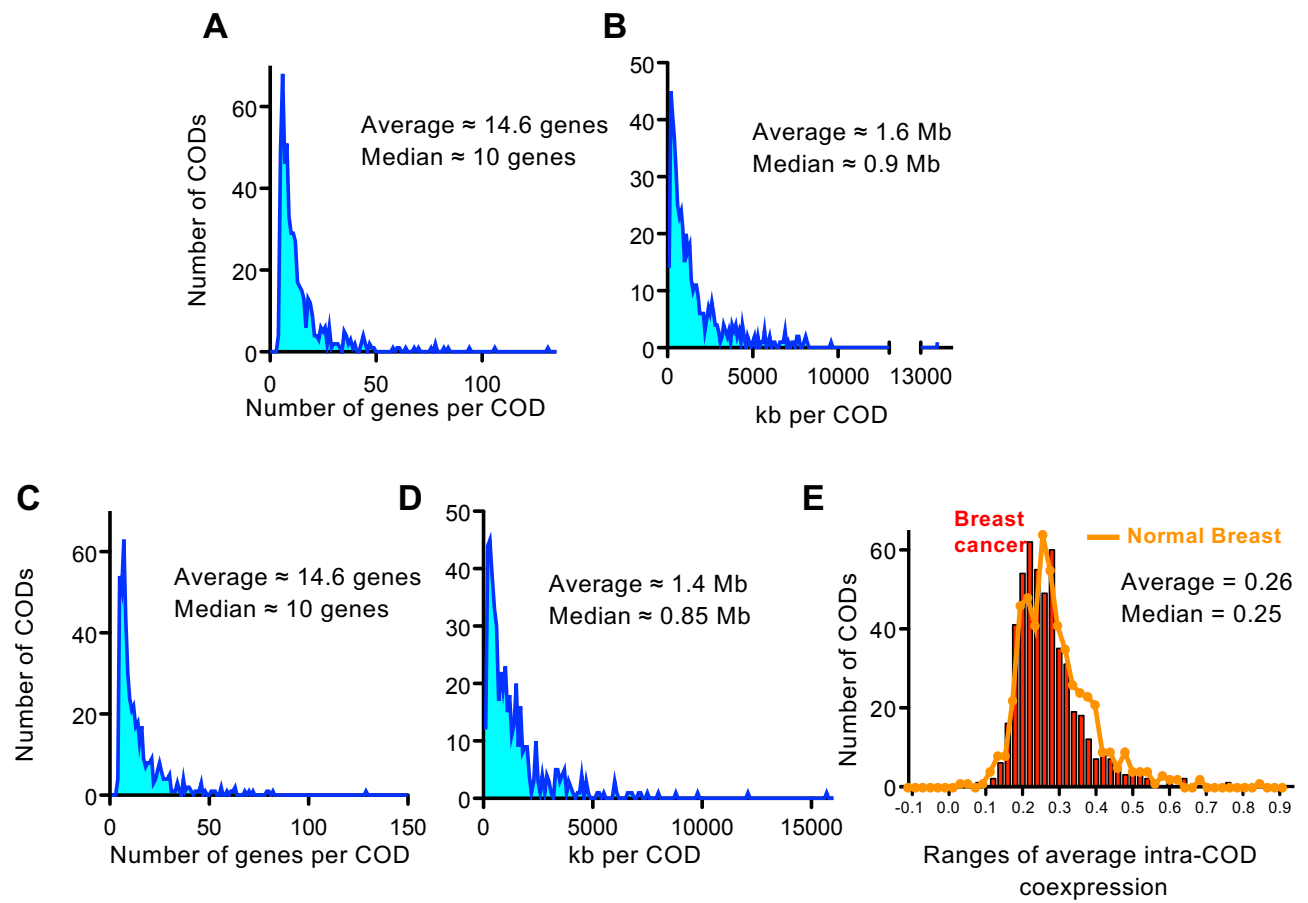

Supplementary figure S5

Supplement: S5 Fig — (A, C) Number of genes per COD distribution in normal (A) and cancer (C) breast samples. (B, D) Size distribution of CODs in normal (B) and cancer (D) breast samples. (E). Distribution of average intra-COD coexpressions of breast cancer CODs. Distribution of the same parameter of normal breast tissue is also plotted for comparison (yellow line). (PDF) [file pcbi.1005708.s005.pdf]

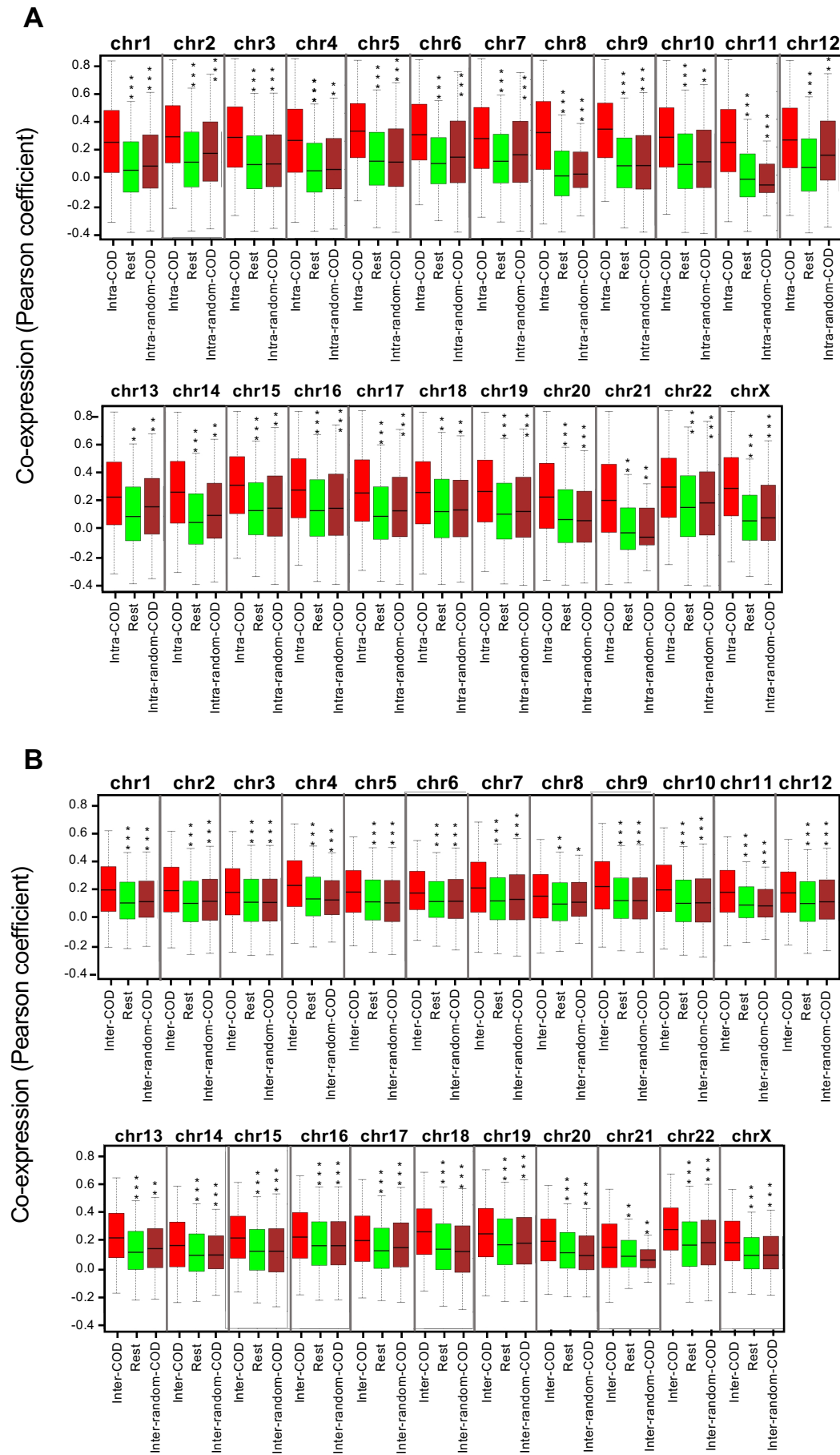

Supplementary Figure S6

Supplement: S6 Fig — (A, B) COD borders were randomized by inverting the chromosomal coordinates from 5′–3′ to 3′–5′ on each chromosome. (A) Box plot of coexpression (Pearson correlation coefficient) of pairs of genes that reside in the same COD (intra-COD), the rest of pairwise gene coexpression (rest), and pairs of genes that reside in the same randomized CODs (Intra-random-COD). (B) Box-plot of coexpression (Pearson correlation coefficient) of pairs of genes that reside in different CODs (inter-COD), the rest of pairwise gene coexpression (rest), and pairs of genes that reside different randomized CODs (Intra-random-COD). ***p < 10−100; **p < 10−10; Bonferroni-corrected p-values of the Mann-Whitney test. (PDF) [file pcbi.1005708.s006.pdf]

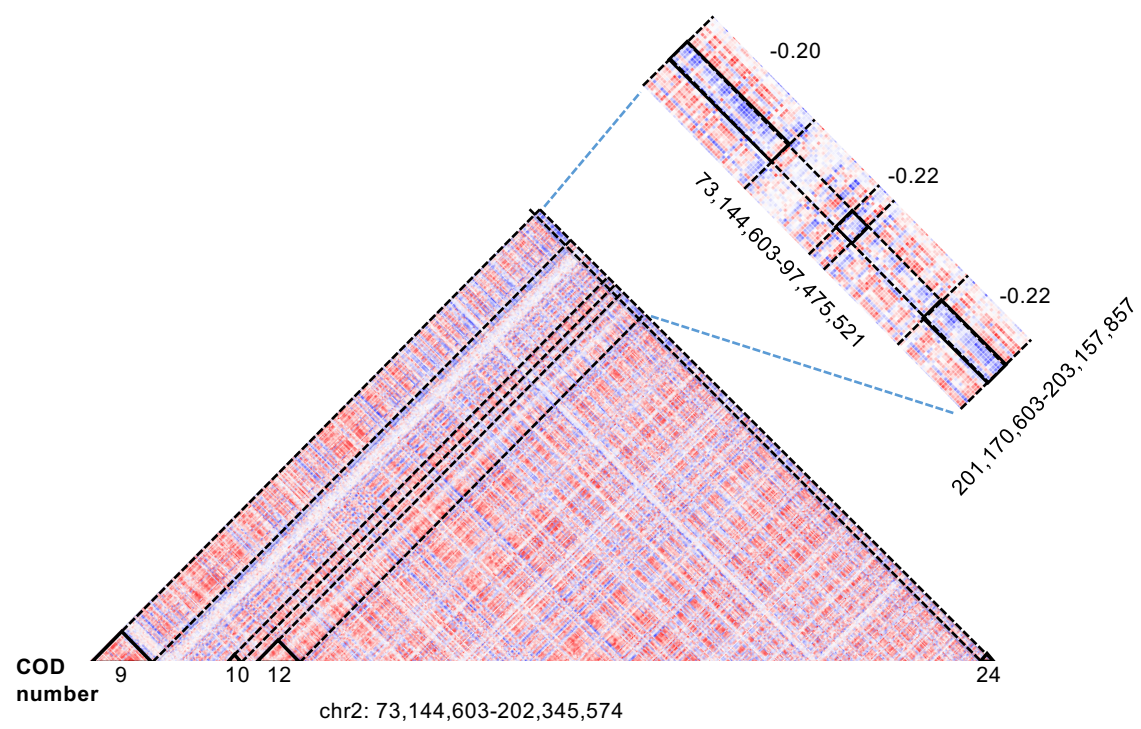

Supplementary Figure S7

Supplement: S7 Fig — Detail of heat map of coexpression of chromosome 2. COD numbers according to S1 Table are provided. Inter-COD regions between CODs 9–24, 10–24, and 12–24 are highlighted, and the average inter-COD coexpression is given. (PDF) [file pcbi.1005708.s007.pdf]

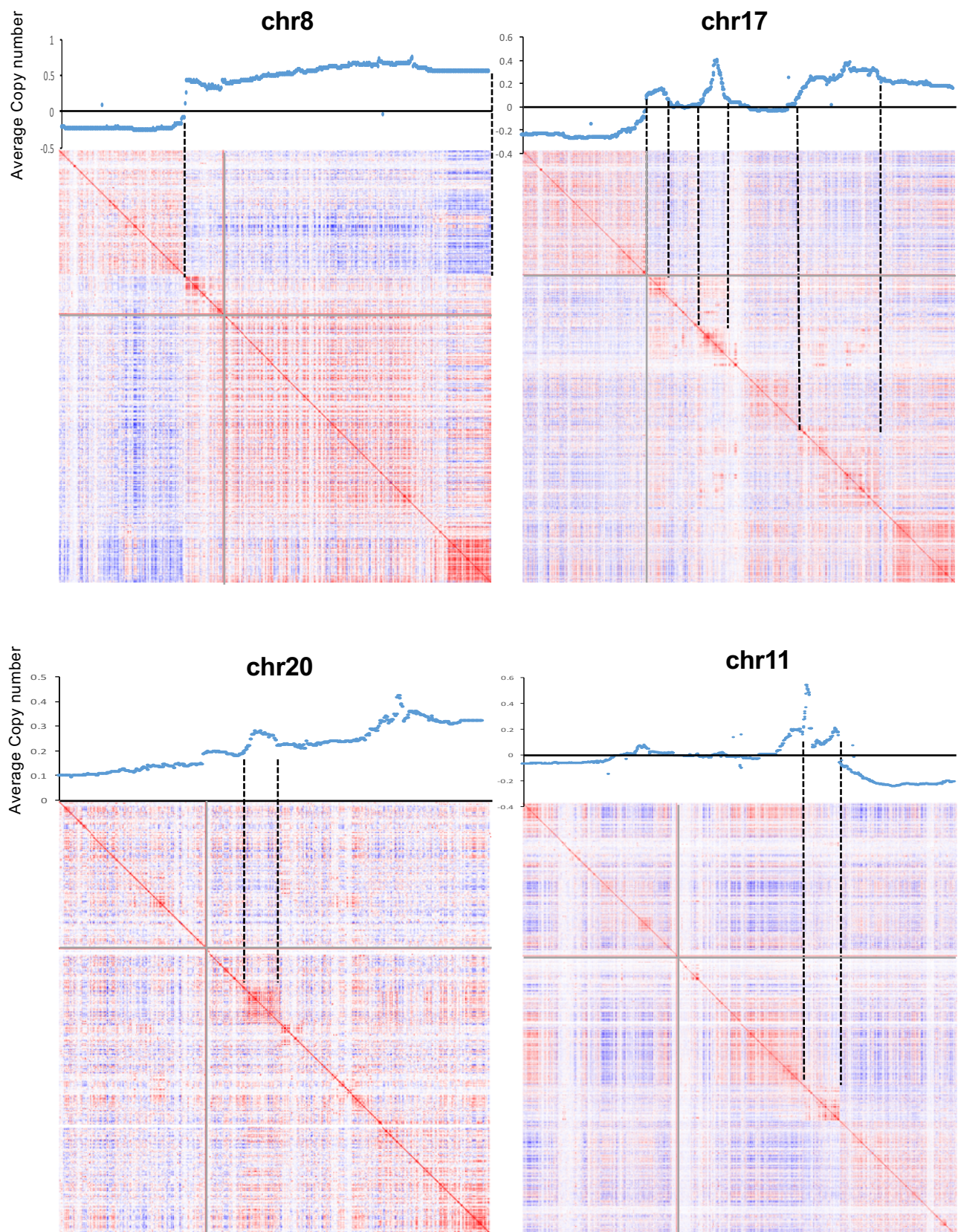

Supplementary Figure S8

Supplement: S8 Fig — The copy number profile in breast cancer samples of chromosomes 8, 11, 17, and 20 were overlaid onto the breast cancer coexpression heat maps. Each pixel of the heat map represents the Pearson coefficient of the correlation between expressions of gene i (columns) and gene j (rows) in 369 breast cancer samples. Coexpression ranges from –1 (blue) to +1 (red). Average gene copy number values (relative linear copy number from Affymetrix SNP6 from TCGA) of the same 369 breast tumor samples were plotted in the chromosomal gene order. Centromeric region is depicted in grey for reference. (PDF) [file pcbi.1005708.s008.pdf]

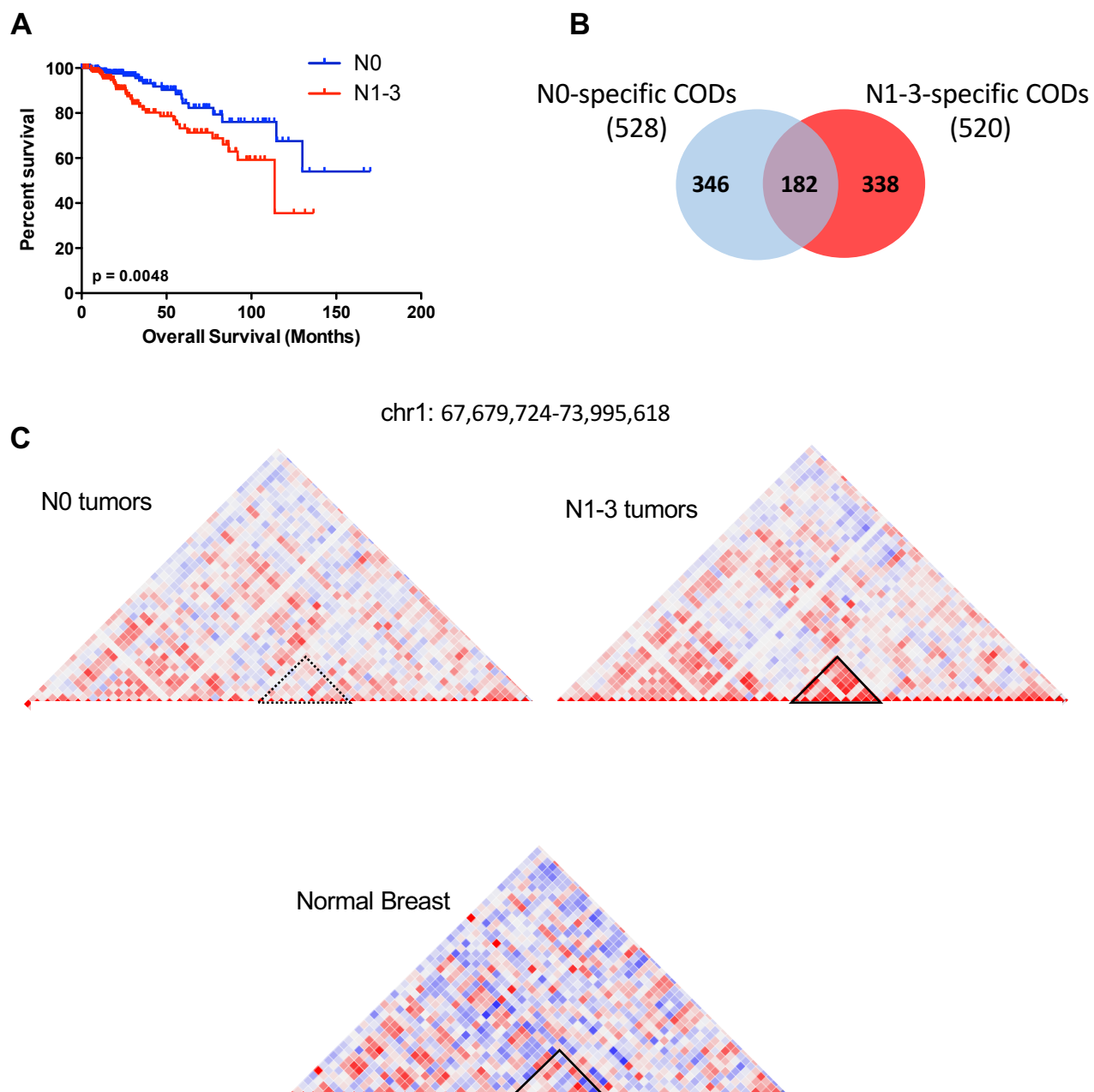

Supplement: S9 Fig — (A) Comparison of Kaplan-Meier survival plots of patients with N0 tumors (n = 180) and patients N1-3 tumors (n = 186). Log-rank test p-value is provided. (B) Overlapping of CODs (at least 80% identical) between N0 and N1-3 tumors. C) Heat maps showing detail of a COD of chromosome 10 that is present in N1-3 tumors and absent in N0 tumors and normal breast. (PDF) [file pcbi.1005708.s009.pdf]

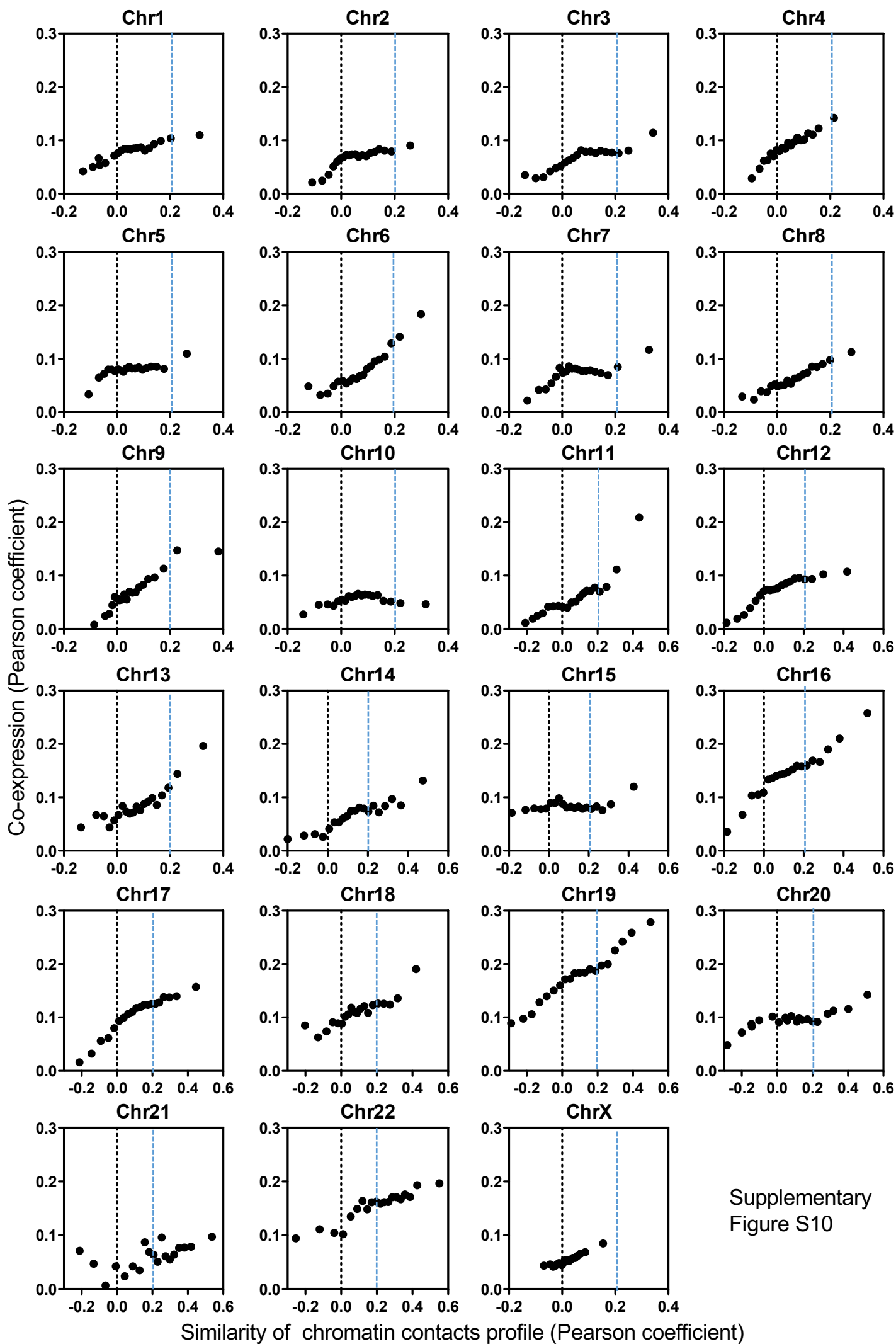

Supplementary  
Figure S10

Supplement: S10 Fig — Chromatin connectivity data of all possible pairs of gene of each chromosome (Pearson correlation matrices of the O/E, see methods) were ranked and grouped into 20 bins with the same number of elements. The average value of connectivity of each bin was then represented against the average coexpression of the corresponding pairs of genes. (PDF) [file pcbi.1005708.s010.pdf]

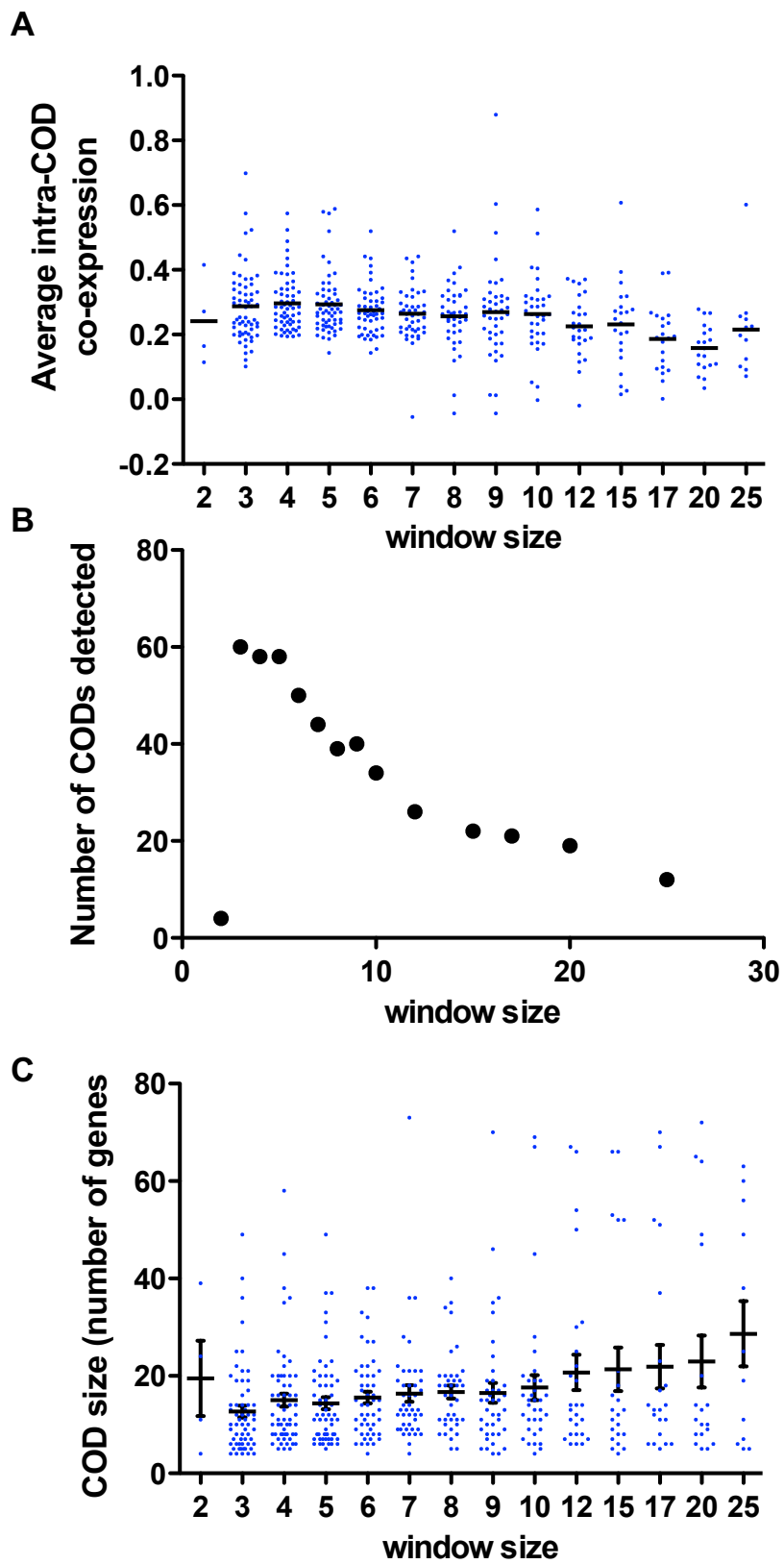

Supplementary  
Figure S11

Supplement: S11 Fig — A) Average intra-CODs coexpression dependence of the w value. B) Variation of the number of CODs detected with different w values. C) Variation of the COD size (in number of genes) with different w values. (PDF) [file pcbi.1005708.s011.pdf]
